# Offering mental health first aid to a person after a potentially traumatic event: a Delphi study to redevelop the 2008 guidelines

**DOI:** 10.1186/s40359-020-00473-7

**Published:** 2020-10-06

**Authors:** Kathryn J. Chalmers, Anthony F. Jorm, Claire M. Kelly, Nicola J. Reavley, Kathy S. Bond, Fairlie A. Cottrill, Judith Wright

**Affiliations:** 1 Centre for Mental Health, Melbourne School of Population and Global Health, University of Melbourne, Parkville, Victoria Australia; 2 Mental Health First Aid Australia, Parkville, Victoria Australia

**Keywords:** Mental health first aid, Trauma, Traumatic event, Prevention, Helping behaviour, Mental illness, Mental health, Delphi method, Expert consensus, Community guidelines

## Abstract

**Background:**

Trauma has a major impact on the mental health and wellbeing of people globally. Friends, family and members of the public are often well positioned to provide initial assistance if someone is experiencing extreme distress following a potentially traumatic event. Expert consensus guidelines for high income, Western countries on how to do this were published in 2008. The aim of the current study was to re-develop these guidelines to ensure they are current and reflect best practice.

**Methods:**

The Delphi consensus method was used to determine which helping statements should be included in the guidelines. Helping statements were derived from a systematic search of literature that considered how a member of the public could help someone experiencing extreme distress following a potentially traumatic event. Two expert panels, comprising 28 mental health professionals with expertise in managing trauma and 26 consumer advocates, rated each statement. Statements were accepted for inclusion in the guidelines if they were endorsed by at least 80% of each panel.

**Results:**

Out of 183 statements, 103 were endorsed as appropriate helping actions in providing assistance to someone experiencing extreme distress following a potentially traumatic event. These statements were used to form the re-developed guidelines.

**Conclusion:**

This study has resulted in a more comprehensive set of guidelines than the original version, with the endorsement of 103 helping actions, compared to 65 previously. The updated guidelines better represent the complexities of experiencing trauma and the considered approach required when providing first aid after a potentially traumatic event. The additional guidance on providing initial assistance, talking about the trauma, offering short-term assistance and seeking appropriate professional help reflects current knowledge. A notable addition is the inclusion of content on how a first aider can assist after a disclosure of abuse. The guidelines are available to the public and will inform future updates of Mental Health First Aid training courses.

## Background

Potentially traumatic events are powerful and distressing experiences that are usually life-threatening or pose a significant threat to a person’s physical or psychological wellbeing. The term “potentially traumatic event” reflects that such events may have little impact on one person but cause severe distress to another. These events may cause anxiety, depression, acute stress disorder (ASD), posttraumatic stress disorder (PTSD), or other diagnosable mental illness.

In 2015, it was estimated that in the past year, 3.6% of the global population experienced an anxiety disorder and 4.4% of the global population experienced a depressive disorder [1]. Anxiety disorders were ranked as the sixth largest contributor to non-fatal health burden and depressive disorders ranked as the largest contributor [1]. In 2010, mental and substance use disorders accounted for 183.9 million disability-adjusted life- years (DALYs) or 7.4% of all DALYs globally [2]. Significant treatment gaps for mental disorders have been described worldwide with professional treatment only received by a minority of those who need it [3–6].

Early assistance or support from family or friends, as well as professional treatment, may help to prevent the onset of mental illness or may minimise the severity of mental illness should it develop. Given this, and that many people do not receive treatment, family and friends can play a role in recognising the symptoms of mental illness, providing support and, if needed, encouraging appropriate professional help. However, Australian research has found that substantial improvements are required in the knowledge and skills needed to help someone experiencing mental illness or a mental health crisis, particularly in specific areas, for example providing support and information to someone experiencing PTSD [7]. The Australian public’s knowledge about anxiety and trauma is also lower than for other mental health conditions like depression [8]. However, with the appropriate skills and knowledge, family and friends are well placed to help someone who experiences a potentially traumatic event [9].

The Mental Health First Aid (MHFA) course was developed to teach members of the public how to recognise when someone is developing a mental health problem or in a crisis and to assist them by providing mental health first aid [10]. Mental health first aid is defined as:

*The help offered to a person developing a mental health problem, experiencing a worsening of an existing mental health problem or in a mental health crisis. The first aid is given until appropriate professional help is received or until the crisis resolves* [11]*.*

Mental health first aid guidelines have been developed to provide recommendations on how one can assist a person with mental health problems (e.g. psychosis [12]), or experiencing a mental health crisis (e.g. engaging in non-suicidal self-injury [13]). All mental health first aid guidelines were developed systematically using the Delphi expert consensus method and were used to inform the content of the 2nd, 3rd and 4th editions of the Australian MHFA course [10, 14, 15], which has spread to over 25 countries [16]. The guidelines were also made available online for the public to access from the MHFA website (https://mhfa.com.au). A study evaluating the impact of the mental health first aid guidelines found that most users who downloaded them subsequently assisted someone using mental health first aid [17].

Evaluation of the MHFA course has found it to be effective in decreasing stigmatising attitudes and increasing helping behaviours towards people with mental health problems [18]. A systematic review of randomised controlled trials of MHFA training, conducted in 2017, supported the effectiveness of MHFA training in improving mental health literacy and support for those with mental health problems up to 6 months after training [19].

Guidelines for supporting a person affected by a traumatic event were developed in 2008 [20, 21]. The current study aims to re-develop these guidelines to ensure content reflects current evidence and best practice. These guidelines are designed for high income, Western countries, as were the 2008 guidelines. Similar guidelines are currently being developed for a number of middle-income countries [22].

Such updates take into account the latest research findings and recommendations for experts in the area of psychological trauma. Previously mental health first aid guidelines for suicidal thoughts and behaviours, non-suicidal self-injury and depression were revised using the Delphi method [13, 23, 24]. These revised guidelines had more detail and recommended more first aid actions than the original guidelines, indicating that regular revision of the full suite of guidelines is ideal.

The Delphi method involves a panel of experts making private, independent ratings of agreement with a series of statements [25]. This expert consensus method provides a systematic way of drawing on the expertise of people working in a particular area [26] and has been used to develop a range of mental health first aid guidelines, including the original trauma guidelines [21]. It allows practice-based evidence to be collected from experts and is a feasible and ethical approach for a study where a randomised controlled trial is not possible. Using this method, expert consensus from panel members located globally can be obtained using an online platform.

This study aimed to use the Delphi method to re-develop guidelines for providing assistance to someone experiencing extreme distress following a potentially traumatic event. Since expertise on providing mental health first aid may come from professional or personal experience, this study required the consensus of panels of consumer advocates (people with lived experience of trauma with subsequent mental health problems) and mental health professionals.

## Methods

Guideline re-development included three stages: literature search, questionnaire development and Delphi consensus survey rounds.

### Literature search

A systematic search was carried out to find statements about how a member of the public can help someone experiencing extreme distress following a potentially traumatic event, including what to do at the site of a potentially traumatic event, how to talk with someone following a potentially traumatic event, what to do if the person discloses abuse, how to offer short-term assistance to the person and how to assist them to seek appropriate professional help if it is needed. Online materials, research publications and books were included in the search for relevant statements. All searches were set to return results published since 2007, as the aim was to find content that had not been covered by the literature search in the original study.

Google search engines of several countries were tested in exploratory searches to identity a combination that provided the highest level of discrete results for the literature search. The search engines identified for use were: Google.com, Google.com.au, Google.co.uk, Google.co.nz, and, Google.ca. The use of additional Google search engines from other countries (e.g. Denmark, Ireland, Sweden and the Netherlands) was found to produce a high level of duplicate results so these search engines were not used. The search terms entered were: “help* trauma past OR present OR current OR experiencing OR friend OR family OR someone”. These terms were chosen because they delivered more relevant results than other combinations. The original study used a single search term “traumatic event”. Using the term ‘trauma’ and excluding the term ‘event’ produced results looking at a broader range of potentially traumatic events. Searches were conducted in private or incognito mode to minimise the influence of Google’s search algorithms. The search settings were adjusted each time to reflect the country of the search engine. Based on previous similar Delphi studies [27], websites appearing in the top 50 results from each search were reviewed. Previous studies have found that the quality of the resources declines after the first 50 results [28]. Overall, 250 websites were reviewed for potential first aid helping actions, duplicate sites were deleted, and relevant statements were found on 32 of these sites. Any links on these websites which the authors thought may contain useful information were followed. A total of 34 online sources resulted, all of which contained relevant statements.

In addition, PsycInfo and PubMed were used to run a title search with the terms ‘trauma’, ‘post traumatic stress’ ‘PTS’ (truncated to include terms such as ‘PTS’ and ‘PTSD’), ‘stress’ (truncated to include terms such as ‘distress’ and ‘stressed’), ‘support’, ‘help’ (truncated as above), ‘assist’, ‘first aid’. The term ‘life support’ was excluded to ensure the return of relevant results. In contrast, the single search term ‘trauma*’ was used in the original study, as attempts to narrow the search had been found to exclude too many relevant results. Searches on these databases returned a total of 2728 results. Any duplicates were deleted and the remaining articles were then screened for relevance. Following this process, 26 articles were deemed relevant. One further article identified in the web search was added for review, resulting in a total of 27 articles. The irrelevant articles were excluded through a tiered screening process, beginning with titles, abstracts and then a full-text review. Of the articles read, ten contained relevant statements.

To locate relevant books, an advanced search of Amazon.com was conducted using the terms ‘trauma’ (truncated as above), ‘help’ and ‘friend’. As in the original study, the Amazon website was chosen because of its extensive coverage of books that included works about mental health aimed at the public. The search differed from the original study, principally with the inclusion of ‘help’ and ‘friend’ as search terms, because this produced the most relevant results when tested. The search returned 39 books, with five books considered relevant. These five books were read, with relevant statements found in one of these books. Irrelevant results included books that were autobiographical in nature, self-help workbooks and clinical manuals.

Existing interventions for responding to traumatic events were also reviewed where possible. Course materials obtained were most commonly aimed at professionals or were focused on policies and procedures recommended for organisations.

### Questionnaire development

Statements derived from the literature search were written up as individual questionnaire items. The first questionnaire was made up of these items, as well as statements from the previous Delphi questionnaires on trauma. Statements included from the previous Delphi were those that were endorsed for the original guidelines, as well as those that were endorsed by 50% or more of both the original panels. These items were reviewed in light of the updated definition of mental health first aid and the first aider’s role, as well as for comprehensibility. Some items were reworded to capture new actions suggested by the literature search or to make them clearer.

In the current study, statements were considered acceptable for inclusion in the questionnaire if the authors agreed that they described how someone could help a person experiencing extreme distress following a potentially traumatic event. Items were required to be relevant to the role of first aider and actionable, as well as being clear in meaning. Adhering to this criteria reduced researcher bias in the selection process. Researchers did not judge the items as this is the role of the experts. Examples of the types of statements included in the questionnaire include:

*The first aider should communicate with the person as an equal, rather than as a superior or expert.*

*If the person experiences flashbacks, the first aider should ask the person how they wish to be supported when these occur*.

Statements were sorted into thematic categories and similar statements were edited to reduce repetition. A working group comprising the authors reviewed this content and edited it to improve clarity, for example by re-wording statements and adding examples. The working group were all researchers with expertise in Delphi methodology and MHFA training programmes. Please see Additional file 1 for a copy of the Round 1 survey.

### Delphi consensus survey rounds

The Delphi method [25] used involved identifying and recruiting panels of experts in the field of psychological trauma. The expert panels then completed online questionnaires, rating each statement according to how important it was that the item be included in the guidelines. A 5-point Likert scale was used (‘essential’, ‘important’, ‘don’t know/depends’, ‘unimportant’ or ‘should not be included’). Statements that achieved substantial consensus as being ‘essential’ or ‘important’ amongst the panellists (80% or more of the members in both panels) were considered to be recommended actions for helping someone after a potentially traumatic event. Questionnaires were presented to panellists via a survey website, Survey Monkey, in three sequential rounds.

#### Panel recruitment

Participants were recruited from high-income, western countries (including Australia, Canada, Ireland, New Zealand, United Kingdom, the United States of America and countries throughout Europe) to join expert panels representing two areas of expertise: consumers (those with lived experience) or professionals. Participant recruitment was restricted to high-income, western countries with similar health care systems because experts from countries that fall outside this criteria approach mental health first aid differently [29]. Many countries require guidelines tailored specifically for their local context. Some of the authors are currently involved in developing similar guidelines for a number of middle-income countries [22].

Panellists were required to have professional experience working in the field of psychological trauma (i.e. as a researcher, clinician, mental health worker), or personal experience with extreme distress following a potentially traumatic event. Prospective professional panellists were identified as experts through their involvement with mental health organisations or professional bodies, while consumer panellists were identified through their advocacy roles.

The 2008 study that developed the original guidelines had aimed to recruit three panels: mental health professionals, consumers and carers [21]. However, recruitment of carers was difficult and there were not enough carers to form their own panel. The carers that were recruited also had lived experience as consumers and thus they were included in the consumer panel instead. Due to these difficulties, as well as challenges in recruitment in more recent Delphi studies [24], it was decided that there would not be a carer panel in the present study.

Professional panellists were recruited through editorial boards of relevant academic journals, mental health advocacy organisations and professional bodies. Offices of organisations providing MHFA in relevant countries were also a source of recruitment. Researchers with published work in the field were directly invited to participate via email and all professionals were also asked to nominate any colleagues who they felt would be appropriate panel members.

Consumer Advocate panellists were recruited through mental health advocacy organisations, including Beyond Blue (Australia), Canadian Mental Health Association, Shine (Ireland), Mental Health Foundation of New Zealand, Scottish Mental Health Welfare Commission, Föreningen Hjärnkoll (Sweden) and Grow in America. Other advocates invited to participate in the consumer panel included public speakers and authors of websites or books offering support and information to those with lived experience of trauma, as well as promoting recovery after a potentially traumatic event. Experts were also asked to nominate anyone else they knew who they felt would be appropriate panel members. The recruitment criteria described ensured that researcher bias was reduced during the panel recruitment stage.

Participants represented global professions in psychological trauma and resided in 10 different countries (see Additional file 5 for further demographics). Approximately half of all participants (54%) had another source of expertise on trauma in addition to their identified expertise, e.g. lived experience or carer experience as well as professional experience. It was not a requirement that participants had prior knowledge on MHFA training courses. Participants were given a brief introduction to how the guidelines would be applied to training courses prior to completing the first survey.

#### Survey rounds

Participants rated the statements included in each questionnaire based on how important they judged them to be in relation to the role of the first aider and the aims of mental health first aid (participant instructions are included in the Additional files 1, 2 and 3).

Questionnaires were analysed using predetermined criteria. This categorised statements into one of three groups that determined whether a statement was endorsed for the guidelines, rejected or re-rated in the following round:

1. Endorsed: statements rated as ‘essential’ or ‘important’ by 80% or more of the members in both panels.
2. Re-rate: statements rated as ‘essential’ or ‘important’ by 70–79.9% of both of the panels, or items rated as ‘essential’ or ‘important’ by 80% or more of one panel, and between 70 and 70.9% by the other panel.
3. Rejected: all other statements were excluded.

In Round 1, panel members were also asked to provide open-ended feedback after each section of the questionnaire. This allowed panellists to suggest helping actions that were not included in the first questionnaire. The authors reviewed this feedback and suggestions that contained original ideas were used to develop new helping statements to be included in the Round 2 questionnaire. Any statements that received feedback suggesting uncertainty in the interpretation of its meaning were re-phrased to make them unambiguous. These were included in the Round 2 questionnaire along with the statements from Round 1 that met the criteria to be re-rated.

The final questionnaire (Round 3) included items that were developed from Round 1 feedback and included for the first time in Round 2, but required re-rating in an additional round. Items that still did not achieve consensus in the final round were rejected from inclusion in the guidelines. See Additional files 1, 2 and 3 for copies of the three surveys.

Following each round, panellists were sent individualised reports containing a summary of the results. The report consisted of a list of statements that had been endorsed for guidelines inclusion, a list of statements that had been rejected, and a list of statements that were to be re-rated in the next survey round. Each report was tailored to include the individual panellist’s rating for each statement, alongside a summary of each panel’s ratings for the statement. This allowed panel members to compare their response to that of the two groups and decide whether to maintain or modify their ratings in the next survey round.

The statements that were endorsed across the three survey rounds were compiled into thematic sections to form the draft guidelines. To improve comprehensibility, statements were re-written as an integrated text and any repetition was deleted. The authors met to finalise structure and wording. The draft of the guidelines was then disseminated to panellists for their final comment and endorsement. At this stage panellists could not suggest new content; however, they were able to provide feedback to improve clarity and reduce ambiguity.

#### Ethical considerations

The University of Melbourne Human Research Ethics Committee approved this research. Informed consent was obtained from all participants by clicking ‘yes’ to a question about informed consent in the Round 1 survey.

## Results

### Participants

Fifty-four people were recruited and completed Round 1. Of these 54 participants, 39 identified as female, 12 identified as male and 3 identified with another term. The average age of participants was 47.8 years (SD = 14.02, range 24–81). Participant characteristics for each panel are presented in Additional file 5. Participants were from Australia, Belgium, Canada, France, Germany, Ireland, Norway, Sweden, the United Kingdom and the United States of America. It was challenging to recruit the required number of participants to each panel to provide stable results. The recruitment criteria were thus altered so it was no longer a requirement that targeted countries had licenced the Mental Health First Aid program. This change sought to include a greater number of participants and trauma experts who were previously excluded, specifically those from countries that were part of Europe-wide trauma associations.

The professional panel comprised 28 experts, most of whom had multiple roles, including 13 mental health support program coordinators/directors, 12 psychological trauma researchers, 9 psychologists, 5 social workers, 4 professors/associate professors, 2 psychiatrists, 2 mental health nurses, 2 occupational therapists and 9 who worked in various other mental health support roles. The consumer panel comprised 26 experts with lived experience. Of the total of 54 panellists in round one, 37 completed all three rounds. The participation of professionals and consumer panellists across the three Delphi survey rounds is shown in Table 1.

**Table 1:** Participation of Delphi panellists in each round

	Round 1	Round 2	Round 3	Retention rate (over 3 rounds)
Professionals	28	23	22	78.6%
Consumer Advocates	26	17	15	57.7%

### Item rating

**Fig. 1:**
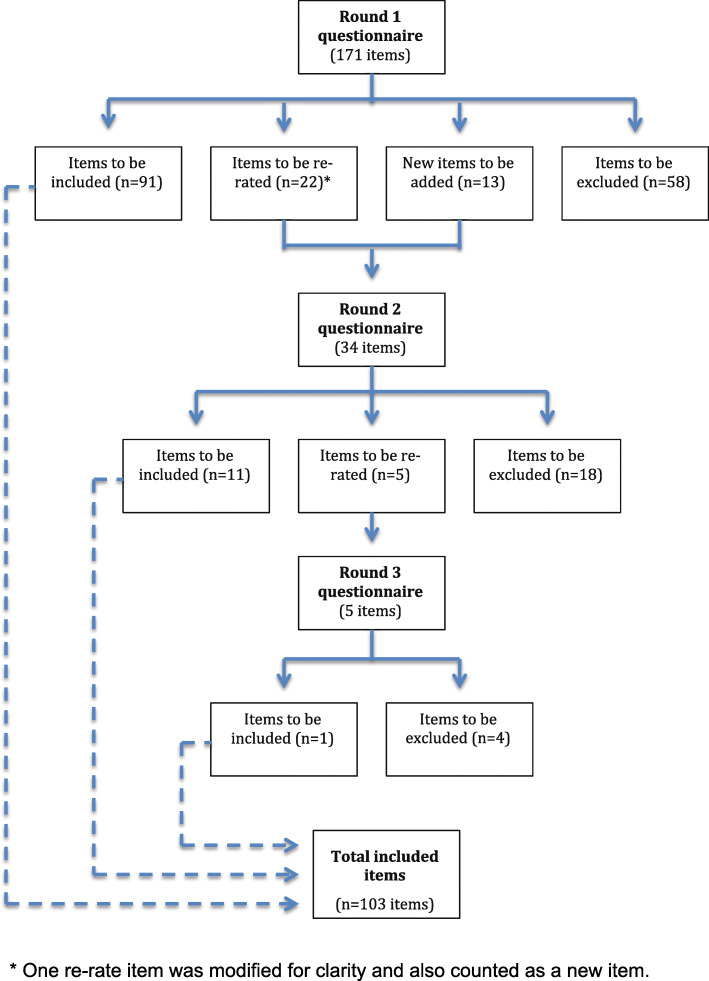
Overview of statements throughout the 3 rounds of questionnaires

A total of 183 items were rated over 3 rounds to yield a total of 103 endorsed items and 80 rejected items. The section headings of the Delphi questionnaire and the number of items that were endorsed and rejected in these sections are shown in Table 2. The rates of inclusion, exclusion and re-rating for each round are shown in Fig. 1: *Overview of statements throughout the 3 rounds of questionnaires*. All statements can be viewed in Additional file 4. The endorsed items formed the basis of the guideline document entitled *Assisting a Person Following a Potentially Traumatic Event: Mental Health First Aid Guidelines (revised 2019)* [30], which is available from the Mental Health First Aid Australia website (mhfa.com.au).

**Table 2:** Sections in the Delphi questionnaire and number of items endorsed and rejected

Section	Topic	Number of items endorsed	Number of items rejected
1	Background information	6	0
2	Actions to be taken at the site of a potentially traumatic event	19	15
3	What to do at the site of a potentially traumatic event where professional helpers are present	3	2
4	Talking about the trauma	31	22
5	Experiences of abuse	19	17
6	Providing support in the weeks & months following a potentially traumatic event	9	7
7	Encouraging professional help	11	11
8	Adolescents	5	6
	TOTAL	103	80

### Difference between panels

Pearson’s r was calculated to determine the correlations of endorsement rates across items between the professional and consumer panels’ ratings. For the 171 items rated in Round 1, the item endorsement rates of the consumer panel and the professional panel were strongly correlated, with a correlation coefficient of .85.

### Difference between the 2008 and 2019 guidelines

A total of 65 items were endorsed for the 2008 guidelines compared to the 103 items endorsed for the current guidelines. There were 34 items endorsed for both the 2008 and 2019 guidelines, as well as 23 items rejected for both. Four items that were endorsed for the 2008 guidelines were not endorsed for the 2019 guidelines, whereas four items that were not endorsed in 2008 were endorsed for the 2019 guidelines. See Additional file 4 for a comparison of item ratings from the 2008 and 2019 studies.

Pearson’s r was calculated to determine the correlations between the two consumer panels, as well as the two professional panels. For the 64 items rated in both years, the endorsement rates of the consumer panel from 2008 were moderately correlated with the consumer panel from 2019, with a correlation coefficient of .44. The endorsement rates of the professional panels from the original and current study were strongly correlated, with a correlation coefficient of .76.

## Discussion

This study aimed to re-develop guidelines published in 2008 for providing assistance to someone experiencing extreme distress following a potentially traumatic event. This was achieved using Delphi expert panels to rate the importance of helping actions for inclusion in the guidelines. The expert panels reached consensus on a range of mental health first aid actions, from what to do at the site of a potentially traumatic event to how to know when the person should seek professional help.

### Comparison with original guidelines

There are some key similarities and differences between the original guidelines and the redeveloped guidelines. The guidelines produced by the current study were formed from 103 endorsed statements. This is a large increase compared with the 65 endorsed in the 2008 study. Of the statements included from the original study, 34 were re-endorsed.

#### Complexity of trauma

The redeveloped guidelines better represent the complexities of experiencing trauma and assisting someone after a potentially traumatic event, by providing a more nuanced approach that reflects current knowledge and balances the needs of the first aider with the needs of the person. Two items that were rejected in the original study and endorsed for inclusion this time were:

*If the person starts to cry, or seems to be trying not to cry, tell them that it is okay to cry or express any feelings they are experiencing.*

*Do not offer religious solace by saying things like “God has reasons”.*

These statements perhaps reflect a growing understanding that everyone react differently to a potentially traumatic event [31, 32], and it is important that they be given permission and space to do so without judgement or pressure.

The present study reflects growing attention, understanding and research in the area of family violence and other types of abuse [33–35] with the inclusion of a specific section on what the first aider should do if someone discloses current or past abuse. Guidance is also given on what the first aider should do if the person dissociates (is ‘spaced out’, ‘shuts down’ or is ‘struggling to communicate’) or experiences a flashback. A short section on supporting an adolescent was also an addition to the redeveloped guidelines, reflecting the understanding that an adolescent may need extra guidance and support [36].

The current guidelines give greater consideration to the limitations and self-care of the mental health first aider, and the challenges of balancing these with providing assistance. For example, one item considers the limitations of the first aider, instructing them that if they do not know what to say to the person, they should admit it. This item was rejected in the 2008 study. A suggested helping action that highlights the complexities of providing assistance while considering self-care is:

*If the person begins to relate details of the abuse that the first aider finds distressing, the first aider should ask the person if they would like assistance finding someone else they can talk to.*

Supporting this action, one professional participant commented on the importance of balancing the needs of the person to be heard (and trying to avoid causing them to feel as if they were being silenced) whilst considering the reality that in some cases the first aider may need to refer to someone else, “They (the first aider) could say they don’t have the expertise to support them and refer them to another service but they have to do it in a way that doesn’t make the person feel as if they shouldn’t talk about it”. Similarly, in the context of the first aider assisting at the scene of a potentially traumatic event, a lived-experience participant stated, “The first aider must also think about their own safety, which has to come first.” A new item to the 2019 guidelines considers what the first aider should do if they find conversations distressing.

A complexity in providing first aid after a potentially traumatic event is identifying when the person should be encouraged to seek professional help. Current advice on the signs and timeframe (4 weeks) in which a person should seek professional help after a potentially traumatic event was similar to the original guidelines.

#### The role of the first aider

The expert panels rejected a small number of items previously endorsed in the original guidelines. These were all items in the section guiding the first aider on what to do at the site of a potentially traumatic event. Possibly the actions suggested by these items were considered to be crossing into a professional role of an emergency worker or a mental health professional, e.g. preserving evidence if a crime has occurred, identifying and meeting the person’s immediate needs (e.g. safety, food, clothing, shelter, medical help or emotional support) and not providing information about the event if the person does not want it. A previous study that redeveloped the mental health first aid depression guidelines also noted similar patterns in terms of actions considered as part of a professional’s role rather than a first aider’s role [24]. In the current study, one lived-experience participant said with regards to provision of information at the site of a potentially traumatic event, “Some info could traumatise further e.g. if they give them information about a possible death of their loved ones…This should really be done by appropriate emergency services not the first aider.” Another possibility is that the current panel members did not consider these statements to be what the first aider should prioritise at the site of potentially traumatic event.

Overall, the increase in the number of recommendations and the greater detail included in the present guidelines may be a reflection of the increase in expertise and research in the area of psychological trauma in the time since the development of the original guidelines. This redevelopment process and its outcomes have reinforced the importance of reviewing guidelines, because changes in the literature and expert opinion can occur rapidly.

### Comparison between ratings of professional and consumer panels

Generally, the professionals and consumers rated items similarly. In Round 1 there were strong correlations between the panels’ ratings. This indicates that overall both panels agreed on what helping actions should be included in the guidelines, and what should be excluded. This included agreement about the importance of the first aider responding to the person in a patient and understanding manner, and actively listening to them without making judgements or assumptions about their experience or reactions. Both panels also agreed on the importance of the first aider knowing some strategies for providing short-term support to the person and what signs and timeframe indicate they should encourage them to seek professional help.

There were a number of items that received a notably different rating (±20%), between the two panels and were endorsed by one panel but not the other. In most cases these items were rejected by the professional panel and were often related to actions that could be perceived as beyond the first aider’s role or control, e.g. *The first aider should try to stay with the person for as long as the person feels it is needed,* and, *The first aider should find out what the person’s immediate needs are (*e.g. *food, clothing, shelter, medical help or emotional support) and attempt to meet them.* Open-ended comments reflected that this action is not always possible.

The starkest difference between the professional and consumer panel opinions was the level of support for this item: *The first aider should encourage the person to seek help from a professional who treats people who have experienced trauma.* This item did not make the guidelines; the consumer panel endorsed it (80.8%) and the professional panel rejected it (57.1%). Possibly here some consumer experts concentrated on the benefit of specialised treatment, whilst a number of professional experts considered that the first aider should encourage any type of professional help to increase the chances that the person is connected with a service.

Although overall there were not large differences between the professional and consumer panels, of the items that were excluded from the guidelines, the two panels rated some quite differently. While 84.6% of consumers endorsed that if the person wants to tell their whole story about the potentially traumatic event, the first aider should give the person enough time to do so, only 64.3% of professionals agreed. The same endorsement percentages were recorded for both panels on another statement, with more consumers than professionals endorsing that the first aider should give the person as much accurate information as they ask for at the site of a potentially traumatic event. Possibly in these statements, many of the professionals saw that this was beyond the role of the first aider and in some cases the role of medical emergency personnel at the scene. The majority of the consumers, on the other hand, perhaps thought that any extra support would be welcomed in these instances.

Unsurprisingly, the consumer panel did not highly endorse support strategies that may be perceived as infringing upon a person’s autonomy. One example was that 92.9% of the professional panel indicated that the first aider should assist the person to make necessary decisions if they appear overwhelmed or indecisive, compared to 73.1% of consumers. Despite this initial difference in Round 1, consensus for this item was achieved in Round 2, with the item endorsed by both panels. Experts from both panels indicated through open-ended comments the importance of protecting the person from additional trauma and keeping them safe whilst allowing them to retain autonomy over all decisions. This perhaps indicates the challenges of striking the right balance as a first aider and the varying reactions that the person may have to any potentially traumatic situation [31].

### Strengths

The inclusion of people with lived experience as a panel is a strength of this study, as it means their expertise is weighted equally with professionals. Use of the Delphi methodology is valuable, as it has enabled the collection of practice-based evidence from experts located in different parts of the world.

A key strength of this study is it provides members of the public access to current and up-to-date content reflecting recommendations from the literature that has been endorsed by expert panellists. The guidelines provide greater depth and direction for the administration of mental health first aid than the 2008 guidelines.

### Weaknesses

Dropout rates, particularly between Rounds 1 and 2, meant that only 58% of consumers participated in all three questionnaire rounds. In comparison, 79% of professionals took part in all three rounds. The time commitments required in the first round (approximately 30–60 min) may have deterred panellists from participation in subsequent rounds. It has been proposed that a minimum of 23 panels members is needed to produces stable results [25]. Due to attrition, the consumer panel only had 15 members by Round 3, a notable limitation, although this limitation was mitigated in that the majority of items (87%) were either rejected or endorsed in Round 1. Another consideration is that panellists may have been asked to rate some items outside their area of expertise and this may have resulted in the exclusion of key actions.

A significant limitation is that these guidelines were developed for use in high-income, Western countries and do not incorporate cultural considerations. The use of these guidelines in countries other than these, or within specific cultural sub-groups, is an area requiring further investigation and consultation with psychological trauma experts from these cultural backgrounds.

#### Future directions

The guidelines received support from the experts involved in this study. Evaluation of the use of these guidelines could explore whether or not they are effective in guiding a first aider to provide assistance to someone experiencing extreme distress following a potentially traumatic event. Any training developed using these guidelines should also be evaluated.

## Conclusions

The mental health first aid guidelines for trauma have been redeveloped to include expert recommendations on the most current and appropriate helping actions. This update has an increased level of detail compared to the previous version of the guidelines, giving more guidance in providing initial assistance, talking about the trauma, assisting after a disclosure of abuse, offering short-term assistance, seeking appropriate professional help, supporting adolescents, as well as important background information about trauma.

The guidelines are available for download on the MHFA website and will be used to inform future revisions of the MHFA course. It is hoped these guidelines will help increase support for people experiencing extreme distress following a traumatic event and provide immediate information for those wishing to assist them.

## Supplementary information

**Additional file 1.** Round 1 Survey. Full survey participants completes in round one. Includes introduction given to participants, consent section and all survey items.

**Additional file 2.** Round 2 Survey. Full survey participants completes in round two. Includes introduction given to participants, consent section and all survey items.

**Additional file 3.** Round 3 Survey. Full survey participants completes in round three. Includes introduction given to participants, consent section and all survey items.

**Additional file 4.** Endorsed and rejected items. Tables presenting all statements that were endorsed as guidelines items and all statements that were rejected. Also presents a comparison between the 2008 study and the 2019 study in terms of statements supported and rejected.

**Additional file 5.** Table 1 Participant characteristics. Tables presenting participant demographics.

## Data Availability

The datasets used and/or analysed during the current study are available from the corresponding author on reasonable request.

## Abbreviations

ASD: Acute stress disorder
DALYs: Disability-adjusted life-years
MHFA: Mental Health First Aid
PTSD: Posttraumatic stress disorder

## References

[1] World Health Organisation (2017). Depression and other common mental disorders: Global Health estimates.

[2] Whiteford HADL, Rehm J, Baxter AJ, Ferrari AJ, Erskine HE, Charlson FJ, Norman RE, Flaxman AD, Johns N, Burstein R (2013). Global burden of disease attributable to mental and substance use disorders: findings from the global burden of disease study 2010. Lancet.

[3] Wang PSA-GE, Alonso J (2007). Worldwide use of mental health services for anxiety, mood and substance disorders: results from 17 countries in the WHO world mental health (WMH) surveys. Lancet.

[4] Demyttenaere KBR, Posada-Villa J (2004). WHO world mental health survey. Prevalence, severity, and unmet need for treatment of mental disorders in the World Health Organization world mental health surveys. JAMA.

[5] Kohn R SS, Levav I, Saraceno B. The treatment gap in mental healthcare. Bull World Health Organ 2004X; 82: 858–866. PMC2623050 15640922

[6] Consortium WMHS (2004). Prevalence, severity, and unmet need for treatment of mental disorders in the World Health Organization world mental health surveys. JAMA.

[7] Rossetto A, Jorm AF, Reavley NJ (2014). Quality of helping behaviours of members of the public towards a person with a mental illness: a descriptive analysis of data from an Australian national survey. Ann General Psychiatry.

[8] Cutler TL, Reavley NJ, Jorm AF (2018). How ‘mental health smart’ are you? Analysis of responses to an Australian broadcasting corporation news webstie quiz. Adv Ment Health.

[9] Charuvastra A, Cloitre M (2008). Social bonds and posttraumatic stress disorder. Ann Review Psychol.

[10] Kitchener BA, Jorm AF, Kelly CM (2017). Mental health first aid manual.

[11] Kitchener BA, Jorm AF, Kelly CM (2015). Mental health first aid international manual.

[12] Langlands RL, Jorm AF, Kelly CM, Kitchener BA (2008). First aid recommendations for psychosis: using the Delphi method to gain consensus between mental health consumers, Carers, and clinicians. Schizophr Bull.

[13] Ross A, Kelly C, Jorm A. Re-development of mental health first aid guidelines for non-suicidal self-injury: a Delphi study. BioMed Central Psychiatry. 2014;14. 10.1186/s12888-014-0236-5 PMC4197339 25134432

[14] Kitchener BA, Jorm AF, Kelly CM (2010). Mental health first aid manual.

[15] Kitchener BA, Jorm AF, Kelly CM (2013). Mental health first aid manual.

[16] Mental Health First Aid Australia. Mental Health First Aid Australia Website Melbourne: Mental Health First Aid Australia; 2019 [cited 2020 02 Jan]. Available from: https://mhfa.com.au.

[17] Hart L, Jorm A, Paxton S, Cvetkovski S. Mental health first aid guidelines: an evaluation of impact following download from the world wide web. Early Intervention Psychiatry. 2012;6. 10.1111/j.1751-7893.2012.00345.x 22379952

[18] Hadlaczky G, Hökby S, Mkrtchian A, Carli V, Wasserman D (2014). Mental health first aid is an effective public health intervention for improving knowledge, attitudes, and behaviour: a meta-analysis. Int Review Psychiatry.

[19] Morgan AJ, Ross A, Reavley NJ (2018). Systematic review and meta-analysis of mental health first aid training: effects on knowledge, stigma, and helping behaviour. PloS one.

[20] Mental Health First Aid Australia (2008). Traumatic events: first aid guidelines for assisting adults.

[21] Kelly CM, Jorm AF, Kitchener BA (2010). Development of mental health first aid guidelines on how a member of the public can support a person affected by a traumatic event: a Delphi study. BioMed Central Psychiatry.

[22] Culturally appropriate approach to improving health outcomes 2020. Available from: https://www.gacd.org/research-projects/mental-health/mh15.

[23] Ross A, Kelly C, Jorm A. Re-development of mental health first aid guidelines for suicidal ideation and behaviour: a Delphi study. BMC Psychiatry. 2014;14. 10.1186/s12888-014-0241-8 PMC4199061 25213799

[24] Bond KS, Cottrill FA, Blee FL, Kelly CM, Kitchener BA, Jorm AF (2019). Offering mental health first aid to a person with depression: a Delphi study to re-develop the guidelines published in 2008. BMC Psychology.

[25] Jones J HD Consensus methods for medical and health services research BMJ 1995; 311. 10.1136/bmj.311.7001.376 PMC2550437 7640549

[26] Jorm AF (2015). Using the Delphi expert consensus method in mental health research. Aust N Z J Psychiatry.

[27] Jorm AF, Ross AM (2018). Guidelines for the public on how to provide mental health first aid: narrative review. BJPsych open.

[28] Kelly CM, Jorm AF, Kitchener BA, Langlands RL. Development of mental health first aid guidelines for suicidal ideation and behaviour: a Delphi study. BMC Psychiatry. 2008;8(1):17. 10.1186/1471-244X-8-17 PMC2324091 18366657

[29] Jorm AF, Ross AM, Colucci E. Cross-cultural generalizability of suicide first aid actions: an analysis of agreement across expert consensus studies from a range of countries and culture. BMC Psychiatry. 2018;18(1):58. 10.1186/s12888-018-1636-8 PMC5831714 29490626

[30] Mental Health First Aid Australia. Assisting a Person Following a Potentially Traumatic Event: Mental Health First Aid Guidelines (revised 2019). Melbourne, 2019.

[31] Bonanno GA, & Mancini, A. D. Beyond resilience and PTSD: Mapping the heterogeneity of responses to potential trauma. Psychological Trauma: Theory Res Practice Policy 2012; 4 (1): 74.

[32] Bonanno GA (2004). Loss, trauma, and human resilience: have we underestimated the human capacity to thrive after extremely aversive events?. Am Psychol.

[33] Anda RFFV, Bremner JD, Walker JD, Whitfield CH, Perry BD, Dube SR, Giles WH (2006). The enduring effects of abuse and related adverse experiences in childhood. Eur Arch Psychiatry Clin Neurosci.

[34] Garcia-Moreno C, & Watts, C. Violence against women: an urgent public health priority. Bulletin of the world health organization 2011: 2. 10.2471/BLT.10.085217 PMC3040024 21346880

[35] Blakemore THJ, Arney F, Parkinson S (2017). The impacts of institutional child sexual abuse: a rapid review of the evidence. Child Abuse Negl.

[36] Nooner KB, Linares LO, Batinjane J, Kramer R A., Silva R., & Cloitre, M. Factors related to posttraumatic stress disorder in adolescence. Trauma Violence Abuse*,* 2012; 13 (3): 153–166. 10.1177/1524838012447698 22665437

