# Supplementary material for: Offering mental health first aid to a person after a potentially traumatic event: a Delphi study to redevelop the 2008 guidelines
Source: BMC Psychol. 2020 Oct 6;8:105. doi: 10.1186/s40359-020-00473-7 (PMC7542436; doi:10.1186/s40359-020-00473-7)
Supplement: Supplementary file 1 — Additional file 1. Round 1 Survey. Full survey participants completes in round one. Includes introduction given to participants, consent section and all survey items. [file 40359_2020_473_MOESM1_ESM.pdf]

Updating the Mental Health First Aid Guidelines for helping someone after a potentially traumatic event

Information about this research

What is this research about?

Researchers from the Centre for Mental Health at the University of Melbourne and Mental Health First Aid Australia and are collaborating to update the mental health first aid guidelines for trauma. The Centre for Mental Health is based at the Melbourne School of Population and Global Health at the University of Melbourne. Mental Health First Aid Australia is a not-for-profit organisation focused on mental health training and research.

The aim of this current research project is to update the mental health first aid guidelines for how a member of the public should give assistance to a person after a potentially traumatic event. These guidelines are being developed for high income Western countries.

There are current mental health first aid guidelines for trauma that were developed in 2008. Given that they are 10 years old, the present study aims to update these guidelines. The guidelines will be available for download on the Mental Health First Aid Australia website (mhfa.com.au) and will be used to inform the Mental Health First Aid course curriculum. These updated guidelines will be applied in training courses and made available to the public.

How will the research be done?

The guidelines will be formed on the basis of expert consensus of participants. Participants will complete online surveys to provide their opinions on a range of strategies for how to help someone experiencing extreme distress following a potentially traumatic event. The strategies that receive a high level of endorsement will be included in the guidelines.

The strategies to be rated in the surveys are obtained from websites, books, fact sheets, brochures, scientific journal articles and training course materials. Some of the statements may seem contradictory or controversial. However, these are included because they reflect the wide range of people's beliefs about intervention and care.

What will you be asked to do?

You will be asked to complete three online surveys over about 4-6 months and the total estimated time commitment is approximately 2-3 hours.

Are there any risks?

Some people may find that reading the statements upsets them, or may remind them of their own experiences. However, in our previous studies using this method only a very small number of people reported feeling upset. Many people in our previous studies have said they felt proud and happy to be able to contribute to a better understanding of how to help others. We advise anyone who finds themselves feeling upset to talk to their support people and decide whether they wish to continue with the questionnaire or stop. In the event that you feel distress and do not know where to go for help, you should contact crisis support. The crisis support number for your country is listed in the information sent to you. This crisis support number can also be found by visiting this website: http://www.cocoonais.com/mental-health-hotlines-worldwide/

Your privacy

We intend to protect your anonymity and the confidentiality of your responses to the fullest possible extent, within the limits of the law. Please note that due to the small number of participants anonymity cannot be guaranteed. Any data we collect from you will be held under password protection and not given to others. We are interested in the consensus views of the panel, rather than the views of individual members. We will only present the results in statistical summary form. We occasionally use participant quotes in published journal articles. When this occurs we do not publish any identifying information with the quote.

Data will be kept securely for a period of 5 years after the last publication based on this data. It will not be deleted until all continued interest in the information ceases.

If you have concerns about the project

This research project has been approved by the Human Research Ethics Committee of The University of Melbourne. If you have any concerns or complaints about the conduct of this research project, which you do not wish to discuss with the research team, you should contact the Manager, Human Research Ethics, Research Ethics and Integrity, University of Melbourne, VIC 3010. Tel: +61 3 8344 2073 or Email: HumanEthics-complaints@unimelb.edu.au. All complaints will be treated confidentially. In any correspondence please provide the name of the research team or the name or ethics ID number of the research project.

The ethics number for this research project is 1851764.1

For more information

You received a Plain Language Statement when you expressed interest in this project. Please refer to this for more details about this study. The Plain Language Statement can also be accessed here: Plain Language Statement.

Consent to participate

* 1. Do you meet the criteria to participate in this study?

You have been invited to participate in this research because you are aged 18 years or over AND:

• Have a lived experience of extreme distress following a potentially traumatic event, feel well enough to participate, AND are engaged in activities that give you a broader exposure to people's experiences of trauma, e.g. you are a member of a consumer advisory or advocacy group, providing peer support to others, etc.

OR

• Are a mental health professional or researcher with at least 5 years experience in the area of psychological trauma.

Do you meet these criteria?

- ○ Yes
- ○ No, please exit the survey now.

It is important for you to know that participation in this study is completely voluntary. You are not under any obligation to participate and you can withdraw at any time.

Best wishes,

The University of Melbourne and Mental Health First Aid Research Team

* 2.

1. I consent to participate in this project, the details of which have been explained to me, and I have been provided with a written plain language statement (see here) to keep.

2. I understand that the purpose of this research is to update the Mental Health First Aid Guidelines for Trauma.

3. I understand that my participation in this project is for research purposes only.

4. I acknowledge that the possible effects of participating in this research project have been explained to my satisfaction.

5. In this project I will be required to complete three online surveys over about 4-6 months.

6. I understand that my participation is voluntary and that I am free to withdraw from this project anytime without explanation or prejudice and to withdraw any unprocessed data that I have provided.

7. I understand that the data from this research will be stored at the University of Melbourne and will be retained for a minimum of 5 years after the last publication using this data. It will not be deleted until all continued interest in the information ceases.

8. I have been informed that the confidentiality of the information I provide will be safeguarded subject to any legal requirements; my data will be password protected and accessible only by the named researchers.

9. I understand that given the small number of participants involved in the study, it may not be possible to guarantee my anonymity, but no personal or identifying data will be published or accessible by anyone outside the research team.

10. I understand that after I consent to participating, any data will be retained by the researcher for a minimum of 5 years after the last publication using this data.

I understand that by submitting this survey I am giving my consent to participate in this study.

◯ Yes, I understand.

◯ I do not consent to participating in this research. Please exit the survey now.

Instructions

Definitions used in this survey:

Mental health first aid is the help provided to a person who is developing a mental health problem, experiencing a worsening of an existing mental health problem or in a mental health crisis. The first aid is given until appropriate professional help is received or the crisis resolves.

The person: someone who is experiencing extreme distress due to a potentially traumatic event.

The first aider: a concerned family member, friend, work colleague or community member, who provides help to a person experiencing extreme distress due to a potentially traumatic event.

Potentially traumatic event: powerful and distressing experiences that are usually life threatening or pose a significant threat to a person's physical or psychological wellbeing.

Many events, past and present, have the potential to be traumatic and not all events commonly perceived as traumatic will cause extreme distress for an individual. Some common examples of events that have the potential to cause trauma include interpersonal violence (including family violence, child abuse, elder abuse, physical or sexual assault, mugging or robbery), accidents (such as traffic or workplace accidents), and witnessing something terrible happen. Mass traumatic events include war, torture, terrorist attacks, mass shootings, and severe weather events (flood, earthquake, hurricane, tsunami, forest and bush fire). Sudden memories of previous events can also cause trauma.

Indirect exposure can also cause trauma, for example witnessing others experience a potentially traumatic event, learning that a potentially traumatic event occurred to someone you know, or repeated or extreme exposure to details of a potentially traumatic events, or multiple potentially traumatic events.

Trauma: an emotional response to a potentially traumatic event. Immediate trauma responses can include shock and denial. Longer term reactions include unpredictable emotions, flashbacks, strained relationships and physical symptoms like headaches or nausea.

Abuse: mistreatment that occurs between people (interpersonal trauma), e.g. emotional, physical or sexual abuse including family violence, child abuse, elder abuse, torture and war crimes.

Professional help: help given by a broad range of relevantly trained health professionals. This could include a mental health professional, GP/family doctor, hospital emergency staff, ambulance officer or paramedic. In this survey these professionals will be called professional helpers.

Emergency services: services that respond to and deal with emergencies when they occur, e.g. emergency medical services (ambulance) or law enforcement (the police).

Instructions (continued)

Please complete the questionnaire by rating each statement according to how important you believe it is for inclusion in the guidelines for providing mental health first aid to a person after a potentially traumatic event.

Please keep in mind that the guidelines will be used by the general public. The statements need to be rated according to their importance for someone WITHOUT a counselling or clinical background. **Please note that we do not seek to replicate areas covered by other existing guidelines.** The final guidelines will direct first aiders to the appropriate mental health first aid guidelines, e.g. depression, suicidal thoughts and panic attack if required (mental health first aid guidelines can be found here).

The majority of statements in the questionnaire pertain to both adults and adolescents. There is also a small section with statements that are additional considerations for adolescents and pertain only to assisting an adolescent.

This questionnaire should take approximately 30-60 minutes to complete. You can complete the survey in two or more sittings. Your answers are saved when you click ‘Next’ at the bottom of a page. This marks your page and you can begin again at a later date on the next page. **Please be aware that once you have logged on and started responding you must complete the questionnaire on the same computer.**

In the next phase of the research you will be asked to complete another two surveys over approximately 6 months. The following two surveys will be considerably shorter and take less time to complete.

Overview of the questionnaire
Section 1: Background Information
Section 2: Actions to be taken at the site of a potentially traumatic event
Section 3: What to do at the site of a potentially traumatic event where professional helpers are present
Section 4: Talking about the trauma
Section 5: Experiences of Abuse
Section 6: Providing support in the weeks and months following a traumatic experience or a disclosure
Section 7: Encouraging professional help
Section 8: Adolescents

Information about you

* 3. What is your name?
(This allows us to determine who has completed the Round 1 survey and is therefore eligible to participate in Round 2. Your name will be deleted from your data when the project is complete).

* 4. How old are you?

* 5. What is your gender?

- ◯ Female
- ◯ Male
- ◯ I identify with another term
- ◯ Do not wish to disclose.

* 6. Please indicate your primary source of expertise.

- ◯ Lived experience
- ◯ Professional

* 7. Please indicate if, in addition to your primary source of expertise, you have other experiences of trauma.

(select all that apply)

- ☐ Person with lived experience
- ☐ Mental health carer or significant support person
- ☐ Mental health professional
- ☐ No other experience of trauma

* 8. Please state the name of the organisation/s you work or volunteer for that make you eligible to participate in this study?

* 9. What is your role within the above organisation/s?

* 10. What country do you live in?

* 11. Are you a Mental Health First Aid Instructor, i.e. do you deliver the Mental Health First Aid course?

- ◯ Yes
- ◯ No

Background information

This section contains statements about what the first aider needs to know about how someone may react to a potentially traumatic event.

Please rate how important (from 'essential' to 'should not be included') you think it is that each statement be included in the guidelines.

Please keep our definitions in mind when responding to this section. You can access the definitions here.

* 12. The first aider should be aware of the initial responses that are common following a potentially traumatic event.

- Essential
- Important
- Don't know/depends
- Unimportant
- Should not be included

* 13. The first aider should know what signs and symptoms can indicate there is a problem after a potentially traumatic event.

- Essential
- Important
- Don't know/depends
- Unimportant
- Should not be included

* 14. The first aider should know the range of symptoms that can occur long-term due to a potentially traumatic event.

- Essential
- Important
- Don't know/depends
- Unimportant
- Should not be included

* 15. The first aider should be aware that people can react very differently following a potentially traumatic event and the first aider should not expect any particular reaction.

- Essential
- Important
- Don't know/depends
- Unimportant
- Should not be included

* 16. The first aider should be aware that there are cultural differences in the way people respond to a potentially traumatic event, e.g. in some cultures, expressing vulnerability or grief around strangers is not considered appropriate.

- Essential
- Unimportant
- Important
- Should not be included
- Don't know/depends

* 17. The first aider should be aware that the person may experience survivor guilt; the feeling that it is unfair that others died or were injured, while they were not.

- Essential
- Unimportant
- Important
- Should not be included
- Don't know/depends

18. Please provide any additional items or comments related to this section.

Actions to be taken at the site of a potentially traumatic event

This section contains statements about what actions the first aider should take at the site of a potentially traumatic event. Some common examples of situations and events that have the potential to cause trauma include interpersonal violence, accidents, witnessing something terrible happen and mass traumatic events. The items in this section concern how a first aider might provide mental health first aid and do not cover physical first aid.

Please rate how important (from ‘essential’ to ‘should not be included’) you think it is that each statement be included in the guidelines.

Please keep our definitions in mind when responding to this section. You can access the definitions here.

* 19. The first aider should determine whether it is safe to approach the person before taking any action, e.g. danger from fire, weapons or debris.

- ◯ Essential
- ◯ Unimportant
- ◯ Important
- ◯ Should not be included
- ◯ Don't know/depends

* 20. The first aider should get medical help for the person if this is needed.

- ◯ Essential
- ◯ Unimportant
- ◯ Important
- ◯ Should not be included
- ◯ Don't know/depends

* 21. The first aider should contact appropriate emergency help, e.g. ambulance, police, fire brigade.

- ◯ Essential
- ◯ Unimportant
- ◯ Important
- ◯ Should not be included
- ◯ Don't know/depends

* 22. If the first aider does not know the person, they should explain what their role is and why they are present.

- ◯ Essential
- ◯ Unimportant
- ◯ Important
- ◯ Should not be included
- ◯ Don't know/depends

* 23. If the first aider does not know the person, they should find out the person’s name and use it when talking to them.

- Essential
- Important
- Don't know/depends
- Unimportant
- Should not be included

* 24. The first aider should try to create a safe environment for the person, e.g. by moving away from traffic, fire or debris.

- Essential
- Important
- Don't know/depends
- Unimportant
- Should not be included

* 25. The first aider should try to appear calm.

- Essential
- Important
- Don't know/depends
- Unimportant
- Should not be included

* 26. The first aider should try not to appear rushed or impatient.

- Essential
- Important
- Don't know/depends
- Unimportant
- Should not be included

* 27. Before taking action, the first aider should observe what help is being provided to the person and whether any additional help is needed.

- Essential
- Important
- Don't know/depends
- Unimportant
- Should not be included

* 28. If the person is already being helped, the first aider should ask the helpers if they need additional assistance or a break.

- Essential
- Important
- Don't know/depends
- Unimportant
- Should not be included

* 29. If the first aider's help is not needed immediately because there are other helpers present, they should remain on site until professional help arrives in case their assistance is needed.

- Essential
- Unimportant
- Important
- Should not be included
- Don't know/depends

* 30. The first aider should find out what the person's immediate needs are (e.g. food, clothing, shelter, medical help or emotional support) and attempt to meet them.

- Essential
- Unimportant
- Important
- Should not be included
- Don't know/depends

* 31. The first aider should be aware of and responsive to the person's comfort and dignity, e.g. by offering the person something to cover themselves with (such as a blanket) or asking bystanders and the media to go away.

- Essential
- Unimportant
- Important
- Should not be included
- Don't know/depends

* 32. The first aider should try to minimise the person's exposure to potentially upsetting sights and sounds, e.g. injured people or flashing lights.

- Essential
- Unimportant
- Important
- Should not be included
- Don't know/depends

* 33. If the person has been a victim of crime, the first aider should consider the possibility that forensic evidence may need to be collected (e.g. evidence on clothing or skin) and should encourage the person to preserve such evidence.

- Essential
- Unimportant
- Important
- Should not be included
- Don't know/depends

* 34. Before assisting the person, the first aider should consider whether they are in a suitable emotional state to provide support to others.

- Essential
- Important
- Don't know/depends
- Unimportant
- Should not be included

* 35. If the first aider feels they are not emotionally able to support the person, they should try to and find somebody else who is.

- Essential
- Important
- Don't know/depends
- Unimportant
- Should not be included

* 36. If the first aider does not feel they are in a suitable emotional state to provide support to others, the first aider should prioritise self-care over support to others.

- Essential
- Important
- Don't know/depends
- Unimportant
- Should not be included

* 37. If the person seems confused, the first aider should attempt to orientate the person to the current place or time.

- Essential
- Important
- Don't know/depends
- Unimportant
- Should not be included

* 38. If the person appears overwhelmed or indecisive, the first aider should assist them to make necessary decisions, e.g. about safety.

- Essential
- Important
- Don't know/depends
- Unimportant
- Should not be included

* 39. The first aider should discourage the person from making any impulsive decisions because they may not be thinking clearly.

- Essential
- Important
- Don't know/depends
- Unimportant
- Should not be included

* 40. The first aider should watch for signs that the person’s physical or mental state is declining, and be prepared to seek emergency medical assistance for them, e.g. an apparently uninjured person may have internal injuries which reveal themselves more slowly, or a person may suddenly become disoriented.

- Essential
- Unimportant
- Important
- Should not be included
- Don't know/depends

* 41. The first aider should try to keep the person updated about what is happening and what is likely to happen next.

- Essential
- Unimportant
- Important
- Should not be included
- Don't know/depends

* 42. The first aider should provide accurate information and admit that they lack information if this is the case.

- Essential
- Unimportant
- Important
- Should not be included
- Don't know/depends

* 43. If the person wants more information about the event, the first aider should try to give them as much accurate information as they ask for.

- Essential
- Unimportant
- Important
- Should not be included
- Don't know/depends

* 44. If the person does not want information about the event, the first aider should not give them any.

- Essential
- Unimportant
- Important
- Should not be included
- Don't know/depends

* 45. If the person's loved ones or friends are not present, the first aider should offer to contact them.

- Essential
- Unimportant
- Important
- Should not be included
- Don't know/depends

* 46. If the person has been separated from loved ones during the potentially traumatic event and wants to be reconnected with them, the first aider should try to help them do so.

- Essential
- Important
- Don't know/depends
- Unimportant
- Should not be included

* 47. The first aider should not make promises they may not be able to keep, e.g. "I'll take you home soon".

- Essential
- Important
- Don't know/depends
- Unimportant
- Should not be included

* 48. If the first aider thinks that someone is not acting in the person's best interests (e.g. trying to get a media interview or stop them reporting to authorities), the first aider should try to protect the person from this.

- Essential
- Important
- Don't know/depends
- Unimportant
- Should not be included

* 49. The first aider should try to stay with the person for as long as the person feels it is needed.

- Essential
- Important
- Don't know/depends
- Unimportant
- Should not be included

50. Please provide any additional items or comments related to this section.

What to do at the site of a potentially traumatic event where professional helpers are present

This section contains statements about what actions the first aider should take at the site of a potentially traumatic event where there are professional helpers present. The items in this section concern how a first aider might provide mental health first aid and do not cover physical first aid.

Please rate how important (from 'essential' to 'should not be included') you think it is that each statement be included in the guidelines.

Please keep our definitions in mind when responding to this section. You can access the definitions here.

* 51. The first aider should follow the directions of professional helpers at the scene.

- ◯ Essential
- ◯ Important
- ◯ Don't know/depends
- ◯ Unimportant
- ◯ Should not be included

* 52. The first aider should not criticise the efforts of professional helpers in front of the person.

- ◯ Essential
- ◯ Important
- ◯ Don't know/depends
- ◯ Unimportant
- ◯ Should not be included

* 53. If the person is asking for information, the first aider should find out from professional helpers what information they are allowed to pass on to the person.

- ◯ Essential
- ◯ Important
- ◯ Don't know/depends
- ◯ Unimportant
- ◯ Should not be included

* 54. In attempting to meet the person's immediate needs, the first aider should not take over the role of professional helpers who may be better able to meet those needs.

- ◯ Essential
- ◯ Important
- ◯ Don't know/depends
- ◯ Unimportant
- ◯ Should not be included

* 55. The first aider should not offer the person food or drink without permission from professional helpers.

- ◯ Essential
- ◯ Important
- ◯ Don't know/depends
- ◯ Unimportant
- ◯ Should not be included

56. Please provide any additional items or comments related to this section.

Talking about the trauma

This section contains statements about what the first aider needs to know when talking with the person about their experiences and feelings following a potentially traumatic event, as well as dealing with challenges that may arise in the discussion. These strategies might be used by the first aider when talking with the person at any stage after a potentially traumatic event. They only cover talking about trauma with the person and do not aim to duplicate other mental health first aid guidelines, e.g. depression.

Please rate how important (from 'essential' to 'should not be included') you think it is that each statement be included in the guidelines.

Please keep our definitions in mind when responding to this section. You can access the definitions here.

* 57. The first aider should communicate with the person as an equal, rather than as a superior or expert.

- ◯ Essential
- ◯ Important
- ◯ Don't know/depends
- ◯ Unimportant
- ◯ Should not be included

* 58. The first aider should try to remain calm when talking with the person, regardless of the person's emotional state.

- ◯ Essential
- ◯ Important
- ◯ Don't know/depends
- ◯ Unimportant
- ◯ Should not be included

* 59. The first aider should reassure the person that their reactions are to be expected under the circumstances.

- ◯ Essential
- ◯ Important
- ◯ Don't know/depends
- ◯ Unimportant
- ◯ Should not be included

* 60. The first aider should show the person they are listening by being patient, even when the person may not be communicating well, e.g. repeating themselves, speaking slowly or unclearly.

- Essential
- Unimportant
- Important
- Should not be included
- Don't know/depends

* 61. If the person begins a sensitive conversation and the first aider does not think it is the ideal place to talk to the person, the first aider should suggest finding an environment likely to be safe, comforting and free of distractions.

- Essential
- Unimportant
- Important
- Should not be included
- Don't know/depends

* 62. If the person begins a sensitive conversation and the first aider does not think it is the ideal time or place to talk to the person, they should still have the conversation rather than delay it.

- Essential
- Unimportant
- Important
- Should not be included
- Don't know/depends

* 63. The first aider should avoid touching the person without their permission, even if touch is usual to their relationship.

- Essential
- Unimportant
- Important
- Should not be included
- Don't know/depends

* 64. The first aider should not tell the person to "snap out of it", "move on" or "focus on the positive" because the impacts of potentially traumatic events cannot be overcome by the person's will power alone.

- Essential
- Unimportant
- Important
- Should not be included
- Don't know/depends

Talking about the person's feelings

* 65. The first aider should encourage the person to talk about their feelings, but only if the person feels ready to do so.

- Essential
- Unimportant
- Important
- Should not be included
- Don't know/depends

* 66. If the person does not wish to talk to the first aider about how they are feeling, they should encourage the person to consider calling a help line or using other community resources.

- Essential
- Unimportant
- Important
- Should not be included
- Don't know/depends

* 67. The first aider should avoid saying things that discourage the person from expressing their feelings, e.g. “don’t cry” or “calm down”.

- Essential
- Unimportant
- Important
- Should not be included
- Don't know/depends

* 68. The first aider should not tell the person how they should be feeling.

- Essential
- Unimportant
- Important
- Should not be included
- Don't know/depends

* 69. The first aider should not tell the person that they "understand how they feel".

- Essential
- Unimportant
- Important
- Should not be included
- Don't know/depends

Talking about the person's experiences

* 70. The first aider should be aware that the person may not remember all the details of a potentially traumatic event.

- ◯ Essential
- ◯ Important
- ◯ Don't know/depends
- ◯ Unimportant
- ◯ Should not be included

* 71. The first aider should express sympathy by saying something like "I'm so sorry about what happened".

- ◯ Essential
- ◯ Important
- ◯ Don't know/depends
- ◯ Unimportant
- ◯ Should not be included

* 72. If the first aider does not know what to say, they should not be afraid to admit it.

- ◯ Essential
- ◯ Important
- ◯ Don't know/depends
- ◯ Unimportant
- ◯ Should not be included

* 73. If the person talks repetitively about the potentially traumatic event, the first aider should listen.

- ◯ Essential
- ◯ Important
- ◯ Don't know/depends
- ◯ Unimportant
- ◯ Should not be included

* 74. If the person shuts the first aider out, the first aider should be patient and ask the person whether they can help in any way.

- ◯ Essential
- ◯ Important
- ◯ Don't know/depends
- ◯ Unimportant
- ◯ Should not be included

* 75. The first aider should tell the person that what happened was not their fault, but only if they know this to be true.

- ◯ Essential
- ◯ Important
- ◯ Don't know/depends
- ◯ Unimportant
- ◯ Should not be included

* 76. If the person feels guilty for something they did or didn't do related to the potentially traumatic event, the first aider should point out to them anything they did right.

- Essential
- Important
- Don't know/depends
- Unimportant
- Should not be included

* 77. The first aider should not say anything to imply that what has happened was the person's fault.

- Essential
- Important
- Don't know/depends
- Unimportant
- Should not be included

* 78. The first aider should not say anything to imply that the person should have reacted or done anything differently at the time of the potentially traumatic event.

- Essential
- Important
- Don't know/depends
- Unimportant
- Should not be included

* 79. The first aider should not avoid talking about the person's experiences.

- Essential
- Important
- Don't know/depends
- Unimportant
- Should not be included

* 80. The first aider should not minimise the person's experience, e.g. "It could have been worse" or "You'll be alright" or "You should be over that by now".

- Essential
- Important
- Don't know/depends
- Unimportant
- Should not be included

* 81. The first aider should not force the person to talk about a potentially traumatic event or their feelings about it.

- Essential
- Important
- Don't know/depends
- Unimportant
- Should not be included

* 82. The first aider should not probe for details of the potentially traumatic event.

- Essential
- Important
- Don't know/depends
- Unimportant
- Should not be included

* 83. The first aider should not offer religious solace by saying things like "God has reasons".

- Essential
- Important
- Don't know/depends
- Unimportant
- Should not be included

* 84. The first aider should not interrupt the person to share their own feelings and experiences.

- Essential
- Important
- Don't know/depends
- Unimportant
- Should not be included

* 85. If the first aider has personally experienced trauma, they should not share the details of this unless asked.

- Essential
- Important
- Don't know/depends
- Unimportant
- Should not be included

* 86. The first aider should not compare the person's experience of trauma with anyone else's.

- Essential
- Important
- Don't know/depends
- Unimportant
- Should not be included

* 87. If the person feels ashamed or guilty about how they reacted during a potentially traumatic event, the first aider should reassure the person that people do not consciously choose how to respond in these situations as these reactions are often automatic and instinctual.

- Essential
- Important
- Don't know/depends
- Unimportant
- Should not be included

* 88. If the person starts to cry, or seems to be trying not to cry, the first aider should tell the person that it is okay to cry or express any feelings they are experiencing.

- Essential
- Unimportant
- Important
- Should not be included
- Don't know/depends

* 89. If the person wants to tell their whole story about the potentially traumatic event, the first aider should give the person enough time to do so.

- Essential
- Unimportant
- Important
- Should not be included
- Don't know/depends

Dealing with challenges during the conversation

* 90. If the person wants to talk about the potentially traumatic event but this is too distressing for the first aider, they should find someone else for the person to talk to.

- Essential
- Unimportant
- Important
- Should not be included
- Don't know/depends

* 91. The first aider should know that behaviour such as withdrawal, irritability and bad temper may be a response to the potentially traumatic event, and should try not to take such behaviour personally.

- Essential
- Unimportant
- Important
- Should not be included
- Don't know/depends

* 92. The first aider should try to be caring, even if they find the person's behaviour is challenging.

- Essential
- Unimportant
- Important
- Should not be included
- Don't know/depends

* 93. If the person seems to be distressed by the conversation, the first aider should ask the person if they need to take a short break.

- Essential
- Important
- Don't know/depends
- Unimportant
- Should not be included

* 94. If the person seems to be 'spaced out', 'shuts down' or is struggling to communicate, the first aider should:

	Essential	Important	Don't know/depends	Unimportant	Should not be included
Not assume that the person does not want to talk.	◯	◯	◯	◯	◯
Encourage the person to move a little, e.g. change their posture, do some stretches.	◯	◯	◯	◯	◯
Ask the person what they need at this moment.	◯	◯	◯	◯	◯
Not force the person to talk about what is distressing them.	◯	◯	◯	◯	◯
Offer to talk to the person at another time.	◯	◯	◯	◯	◯

* 95. If the person experiences flashbacks, the first aider should ask the person how they wish to be supported when these occur.

- Essential
- Important
- Don't know/depends
- Unimportant
- Should not be included

* 96. If the person experiences a flashback the first aider should:

	Essential	Important	Don't know/depends	Unimportant	Should not be included
Tell the person that even though it seems real it is not actually happening.	○	○	○	○	○
Remind the person that they are safe.	○	○	○	○	○
Call attention to the here and now, e.g. referencing the immediate surroundings.	○	○	○	○	○
Offer a glass of water.	○	○	○	○	○
Avoid sudden movements or anything that may startle them.	○	○	○	○	○
Avoid touching them.	○	○	○	○	○

97. Please provide any additional items or comments related to this section.

Experiences of abuse

This section contains statements about what the first aider needs to know when talking with the person following a disclosure of abuse. Abuse for the purpose of this survey includes events that occur between people (interpersonal trauma), e.g. emotional, physical or sexual abuse including family violence, child abuse, elder abuse, torture and war crimes.

Please rate how important (from 'essential' to 'should not be included') you think it is that each statement be included in the guidelines.

Please keep our definitions in mind when responding to this section. You can access the *definitions here.*

* 98. If the person discloses any abuse associated with criminal activity, the first aider should:

	Essential	Important	Don't know/depends	Unimportant	Should not be included
Encourage the person to report it to the police, if it is safe to do so.	○	○	○	○	○
Report it to the police, if it is safe to do so.	○	○	○	○	○
Encourage the person to seek help from an appropriate support service.	○	○	○	○	○

* 99. If the first aider reports any criminal activity to the police, they should let the person know beforehand.

- ○ Essential
- ○ Important
- ○ Don't know/depends
- ○ Unimportant
- ○ Should not be included

* 100. The first aider should be aware of any local mandatory reporting laws.

- ○ Essential
- ○ Important
- ○ Don't know/depends
- ○ Unimportant
- ○ Should not be included

* 101. The first aider should be aware that the person who has experienced abuse may not trust easily and may be reluctant to seek support from others.

- Essential
- Important
- Don't know/depends
- Unimportant
- Should not be included

* 102. If the person discloses abuse, the first aider should listen to them and not feel that they have to provide solutions or advice.

- Essential
- Important
- Don't know/depends
- Unimportant
- Should not be included

* 103. If the person discloses that they are being abused, the first aider should:

	Essential	Important	Don't know/depends	Unimportant	Should not be included
Encourage the person to tell the perpetrator to stop , if it is safe to do so.	◯	◯	◯	◯	◯
Encourage the person to tell the perpetrator how it is affecting them, if it is safe to do so.	◯	◯	◯	◯	◯

* 104. If the first aider sees physical signs of abuse (e.g. repeated bruising), they should discuss their concerns with the person.

- Essential
- Important
- Don't know/depends
- Unimportant
- Should not be included

* 105. If the first aider expresses their concerns about signs of physical abuse and the person dismisses them, the first aider should be aware that it does not necessarily mean that their concerns are misplaced.

- Essential
- Important
- Don't know/depends
- Unimportant
- Should not be included

* 106. If the person dismisses the first aider's concerns, and the first aider sees possible signs of abuse again in the future, they should raise their concerns with the person again.

- ◯ Essential
- ◯ Important
- ◯ Don't know/depends
- ◯ Unimportant
- ◯ Should not be included

* 107. If the first aider expresses their concerns about signs of physical abuse and the person dismisses them and becomes angry, the first aider should apologise to the person.

- ◯ Essential
- ◯ Important
- ◯ Don't know/depends
- ◯ Unimportant
- ◯ Should not be included

* 108. If the first aider is concerned that the person is at risk of harm from someone else, they should:

	Essential	Important	Don't know/depends	Unimportant	Should not be included
Work with the person to identify what steps to take next to keep them safe.	◯	◯	◯	◯	◯
Offer options to keep the person safe, e.g. helping them find alternative accommodation.	◯	◯	◯	◯	◯
Assist the person to identify other people who can provide support.	◯	◯	◯	◯	◯
Encourage the person to call the police and report the situation.	◯	◯	◯	◯	◯
Offer to call the police and report the situation.	◯	◯	◯	◯	◯
Encourage the person to call an appropriate helpline, e.g. family violence helpline.	◯	◯	◯	◯	◯
Offer to call an appropriate helpline on behalf of the person, e.g. family violence helpline.	◯	◯	◯	◯	◯
Not do anything that places themselves at risk.	◯	◯	◯	◯	◯

* 109. If the person asks the first aider not to tell anyone about the abuse they have experienced, the first aider should:

	Essential	Important	Don't know/depends	Unimportant	Should not be included
Respect their wishes.	○	○	○	○	○
Respect their wishes, unless they are at risk of immediate harm.	○	○	○	○	○
Respect their wishes, unless the abuse is likely to be a criminal offence.	○	○	○	○	○

* 110. If the first aider thinks they need to share any of the information the person has told them in order to keep the person safe, they should:

	Essential	Important	Don't know/depends	Unimportant	Should not be included
Consider the possible repercussions for the person, e.g. their safety or relationships.	○	○	○	○	○
Get the person's agreement before doing so.	○	○	○	○	○
Tell the person before doing so.	○	○	○	○	○

* 111. If the person discloses abuse that happened in the past, the first aider should:

	Essential	Important	Don't know/depends	Unimportant	Should not be included
Tell the person that they believe them.	○	○	○	○	○
Not express any feelings of disbelief.	○	○	○	○	○
Thank the person for telling them.	○	○	○	○	○

* 112. If the person begins to relate details of the abuse that the first aider finds distressing, the first aider should protect themselves by:

	Essential	Important	Don't know/depends	Unimportant	Should not be included
Encouraging the person to talk about other things.	○	○	○	○	○
Encouraging the person to talk to a support service rather than the first aider.	○	○	○	○	○

113. Please provide any additional items or comments related to this section.

Providing support in the weeks & months following a potentially traumatic event

This section contains statements about what the first aider needs to know about providing support to the person following a potentially traumatic event. It covers what the first aider can do in the weeks and months following a traumatic event but does not cover ongoing support. These statements are specific to trauma and do not duplicate other mental health first aid guidelines, e.g. depression (mental health first aid guidelines can be found here).

Please rate how important (from ‘essential’ to ‘should not be included’) you think it is that each statement be included in the guidelines.

Please keep our definitions in mind when responding to this section. You can access the definitions here.

Being supportive and understanding

* 114. The first aider should be aware that even weeks or months following a potentially traumatic event, the person might have good days and bad days and that there is no timeline for what can be expected.

- Essential
- Important
- Don't know/depends
- Unimportant
- Should not be included

* 115. The first aider should tell the person that everyone deals with potentially traumatic events at their own pace.

- Essential
- Important
- Don't know/depends
- Unimportant
- Should not be included

* 116. The first aider should tell the person that it is possible to recover from potentially traumatic events.

- Essential
- Important
- Don't know/depends
- Unimportant
- Should not be included

* 117. The first aider should tell the person about any sources of information available for survivors, e.g. information sessions, fact sheets and phone numbers for information lines.

- Essential
- Unimportant
- Important
- Should not be included
- Don't know/depends

* 118. The first aider should discourage the person from making any major life decisions or big life changes, if at all possible.

- Essential
- Unimportant
- Important
- Should not be included
- Don't know/depends

* 119. The first aider should be aware that the person may be more sensitive to events or stress that can seem minor to others.

- Essential
- Unimportant
- Important
- Should not be included
- Don't know/depends

* 120. The first aider should be aware that there are many things that can remind the person of the trauma and cause distress.

- Essential
- Unimportant
- Important
- Should not be included
- Don't know/depends

* 121. The first aider should encourage the person to talk about how the trauma has affected their relationships.

- Essential
- Unimportant
- Important
- Should not be included
- Don't know/depends

* 122. The first aider should be aware that the person may suddenly or unexpectedly remember details of the event and may need additional support at this time.

- Essential
- Unimportant
- Important
- Should not be included
- Don't know/depends

* 123. The first aider should be aware that anniversaries of past potentially traumatic events and media reports of similar experiences can remind the person of the trauma and they may need extra support around these times.

- Essential
- Important
- Don't know/depends
- Unimportant
- Should not be included

Encouraging other supports: family, friends and community

* 124. The first aider should encourage the person to share their **reactions** with people who they think will be supportive.

- Essential
- Important
- Don't know/depends
- Unimportant
- Should not be included

* 125. The first aider should encourage the person to share their **memories** with people who they think will be supportive.

- Essential
- Important
- Don't know/depends
- Unimportant
- Should not be included

* 126. The first aider should encourage the person to use supports that are specific to their type of potentially traumatic event, e.g. veteran services, victims of crime.

- Essential
- Important
- Don't know/depends
- Unimportant
- Should not be included

* 127. If the first aider knows someone else who has had a similar experience, the first aider should suggest to the person that they might speak to them.

- Essential
- Important
- Don't know/depends
- Unimportant
- Should not be included

* 128. If the person has experienced a potentially traumatic event that is receiving media coverage, the first aider should encourage the person to limit their exposure to this media coverage.

- Essential
- Important
- Don't know/depends
- Unimportant
- Should not be included

* 129. If the person has experienced a potentially traumatic event that is receiving media coverage, the first aider should offer to keep track of new or important information so the person does not need to monitor it constantly.

- Essential
- Important
- Don't know/depends
- Unimportant
- Should not be included

130. Please provide any additional items or comments related to this section.

Encouraging professional help

This section contains statements about what the first aider needs to know about encouraging the person to seek professional help.

Please rate how important (from 'essential' to 'should not be included') you think it is that each statement be included in the guidelines.

Please keep our definitions in mind when responding to this section. You can access the definitions here.

* 131. The first aider should be aware of the sorts of professional help that are available for people who have experienced trauma.

- Essential
- Important
- Don't know/depends
- Unimportant
- Should not be included

* 132. The first aider should encourage the person to seek help from a professional who treats people who have experienced trauma.

- Essential
- Important
- Don't know/depends
- Unimportant
- Should not be included

When should the person seek professional help?

* 133. The first aider should encourage the person to seek professional help if the post-trauma symptoms are interfering with their usual activities for:

	Essential	Important	Don't know/depends	Unimportant	Should not be included
2 weeks or more.	◯	◯	◯	◯	◯
4 weeks or more.	◯	◯	◯	◯	◯

* 134. The first aider should encourage the person to seek professional help if they feel very upset or fearful for:

	Essential	Important	Don't know/depends	Unimportant	Should not be included
2 weeks or more.	◯	◯	◯	◯	◯
4 weeks or more.	◯	◯	◯	◯	◯

* 135. The first aider should encourage the person to seek professional help if they are unable to escape intense ongoing distressing feelings for:

	Essential	Important	Don't know/depends	Unimportant	Should not be included
2 weeks or more.	◯	◯	◯	◯	◯
4 weeks or more.	◯	◯	◯	◯	◯

* 136. The first aider should encourage the person to seek professional help if they act very differently after the potentially traumatic event for:

	Essential	Important	Don't know/depends	Unimportant	Should not be included
2 weeks or more.	◯	◯	◯	◯	◯
4 weeks or more.	◯	◯	◯	◯	◯

* 137. The first aider should encourage the person to seek professional help if their important relationships are suffering as a result of the trauma (e.g. if they withdraw from their family or friends) for:

	Essential	Important	Don't know/depends	Unimportant	Should not be included
2 weeks or more.	◯	◯	◯	◯	◯
4 weeks or more.	◯	◯	◯	◯	◯

* 138. The first aider should encourage the person to seek professional help if they feel jumpy or have nightmares because of or about the trauma for:

	Essential	Important	Don't know/depends	Unimportant	Should not be included
2 weeks or more.	◯	◯	◯	◯	◯
4 weeks or more.	◯	◯	◯	◯	◯

* 139. The first aider should encourage the person to seek professional help if they can't stop thinking about the trauma for:

	Essential	Important	Don't know/depends	Unimportant	Should not be included
2 weeks or more.	◯	◯	◯	◯	◯
4 weeks or more.	◯	◯	◯	◯	◯

* 140. The first aider should encourage the person to seek professional help if they are unable to enjoy life at all as a result of the trauma for:

	Essential	Important	Don't know/depends	Unimportant	Should not be included
2 weeks or more.	◯	◯	◯	◯	◯
4 weeks or more.	◯	◯	◯	◯	◯

* 141. The first aider should encourage the person to seek professional help if they misuse alcohol or other drugs to deal with the trauma:

	Essential	Important	Don't know/depends	Unimportant	Should not be included
for 2 weeks or more.	◯	◯	◯	◯	◯
for 4 weeks or more.	◯	◯	◯	◯	◯
at any time.	◯	◯	◯	◯	◯

* 142. The first aider should be aware that most people recover from trauma without needing professional help.

- ◯ Essential
- ◯ Important
- ◯ Don't know/depends
- ◯ Unimportant
- ◯ Should not be included

143. Please provide any additional items or comments related to this section.

Adolescents

This section contains statements about what the first aider needs to know when the person is an adolescent. These are *additional considerations* that only apply to adolescents.

Please rate how important (from 'essential' to 'should not be included') you think it is that each statement be included in the guidelines.

Please keep our definitions in mind when responding to this section. You can access the definitions here.

* 144. The first aider should be aware of the ways in which an adolescent may respond differently to a potentially traumatic event compared to an adult.

- Essential
- Important
- Don't know/depends
- Unimportant
- Should not be included

* 145. If the first aider does not have an ongoing role in the adolescent's life, the first aider should ask them if they have a trusted adult who can support them.

- Essential
- Important
- Don't know/depends
- Unimportant
- Should not be included

* 146. If the adolescent does not have a trusted and appropriate adult to talk to, the first aider should connect them with an appropriate service, e.g. a community program, health centre or help line.

- Essential
- Important
- Don't know/depends
- Unimportant
- Should not be included

* 147. If appropriate to the relationship, the first aider should contact the adolescent's school about any additional support they may need.

- Essential
- Important
- Don't know/depends
- Unimportant
- Should not be included

* 148. If the adolescent has experienced a potentially traumatic event that is receiving media coverage, the first aider should try to limit their exposure to this media coverage.

- Essential
- Important
- Don't know/depends
- Unimportant
- Should not be included

* 149. The first aider should encourage the adolescent to use face-to-face contact with friends rather than through social media.

- Essential
- Important
- Don't know/depends
- Unimportant
- Should not be included

* 150. The first aider should not hide information from the adolescent in an attempt to protect them.

- Essential
- Important
- Don't know/depends
- Unimportant
- Should not be included

* 151. If the adolescent does not want to talk about what has occurred, the first aider should:

	Essential	Important	Don't know/depends	Unimportant	Should not be included
Let the adolescent know that they are ready to talk if the adolescent wants to.	○	○	○	○	○
Ask the adolescent if there is someone else they would prefer to talk to.	○	○	○	○	○
Encourage the adolescent to talk about their feelings rather than about what has occurred.	○	○	○	○	○

152. Please provide any additional items or comments related to this section.

Thank you!

Thank you for sharing your expertise and time with us.

If anything in this survey has caused you distress and you would like to talk with someone about it you can contact the appropriate crisis help line for your country. The help line number for your country can be found in the information provided to you by researchers or by visiting this website:
http://www.cocoonais.com/mental-health-hotlines-worldwide/
