# Supplementary material for: Offering mental health first aid to a person after a potentially traumatic event: a Delphi study to redevelop the 2008 guidelines
Source: BMC Psychol. 2020 Oct 6;8:105. doi: 10.1186/s40359-020-00473-7 (PMC7542436; doi:10.1186/s40359-020-00473-7)
Supplement: Supplementary file 2 — Additional file 2. Round 2 Survey. Full survey participants completes in round two. Includes introduction given to participants, consent section and all survey items. [file 40359_2020_473_MOESM2_ESM.pdf]

ROUND 2: Mental Health First Aid Guidelines for helping someone after a potentially traumatic event

Aim of this research

The aim of this current research project is to update the mental health first aid guidelines for how a member of the public should give assistance to a person after a potentially traumatic event. The previously developed mental health first aid guidelines for trauma were created in 2008.

Your role in Round 2

Your task is to rate the statements presented in this questionnaire according to how important you believe they are for providing guidance to adults assisting a person after a potentially traumatic event.

How this questionnaire was developed

Statements in this questionnaire were developed from two sources:

1. Items for re-rating: These are items from Round 1 that did not receive a level of consensus that was either high enough to be clearly endorsed or low enough to be clearly rejected.

2. *New items:* These items have been developed from comments made by participants in Round 1. In some cases they are items that have been modified from Round 1 based on comments made by participants.

Items that are new are labelled "(New Item)". All items without this label are items for re-rating from Round 1.

It is important to note that we do not necessarily agree with these statements; we have included them because we do not believe that we should decide what the best practice is in this area. Rather, we have invited you to be a member of the expert panel to help develop a set of guidelines that reflect current expert opinion.

Instructions

Please complete the questionnaire by rating each statement according to how important you believe it is for inclusion in guidelines for how a member of the public should give assistance to a person after a potentially traumatic event. This questionnaire should take approximately 10 minutes to complete. If necessary, you can complete the survey in two or more sittings. Your answers are saved when you click ‘Next’ at the bottom of a page. This marks your page and you can begin again at a later date on the next page. **Please be aware that once you have logged on and started responding you must complete the questionnaire on this same computer.**

Your privacy
We intend to protect your anonymity and the confidentiality of your responses to the fullest possible extent, within the limits of the law. Please note that due to the small number of participants anonymity cannot be guaranteed. Any data we collect from you will be held under password protection and not given to others. We are interested in the consensus views of the panel, rather than the views of individual members. We will only present the results in statistical summary form. We occasionally use participant quotes in published journal articles. When this occurs we do not publish any identifying information with the quote.

Data will be kept securely for a period of 5 years after the last publication based on this data. It will not be deleted until all continued interest in the information ceases.

If you have concerns about the project
This research project has been approved by the Human Research Ethics Committee of The University of Melbourne. If you have any concerns or complaints about the conduct of this research project, which you do not wish to discuss with the research team, you should contact the Manager, Human Research Ethics, Research Ethics and Integrity, University of Melbourne, VIC 3010. Tel: +61 3 8344 2073 or Email: HumanEthics-complaints@unimelb.edu.au. All complaints will be treated confidentially. In any correspondence please provide the name of the research team or the name or ethics ID number of the research project.

The ethics number for this research project is 1851764.1

For more information
You received a Plain Language Statement when you expressed interest in this project. Please refer to this for more details about this study. You may also contact Research Officer, Kathryn Chalmers if you require further information: kchalmers1@unimelb.edu.au.

It is important for you to know that participation in this study is completely voluntary. You are not under any obligation to participate and you can withdraw at any time. Submitting your questionnaire is an indication of your understanding of this and your consent to participate in the study.

We would like to thank you for your time and effort and encourage you to provide us with feedback on this process.

Best Wishes,

The University of Melbourne and Mental Health First Aid Australia Research Team

ROUND 2: Mental Health First Aid Guidelines for helping someone after a potentially traumatic event

Instructions

Definitions used in this survey:

Mental health first aid is the help provided to a person who is developing a mental health problem, experiencing a worsening of an existing mental health problem or in a mental health crisis. The first aid is given until appropriate professional help is received or the crisis resolves.

The person: someone who is experiencing extreme distress due to a potentially traumatic event.

The first aider: a concerned family member, friend, work colleague or community member, who provides help to a person experiencing extreme distress due to a potentially traumatic event.

Potentially traumatic event: powerful and distressing experiences that are usually life threatening or pose a significant threat to a person's physical or psychological wellbeing.

Many events, past and present, have the potential to be traumatic and not all events commonly perceived as traumatic will cause extreme distress for an individual. Some common examples of events that have the potential to cause trauma include interpersonal violence (including family violence, child abuse, elder abuse, physical or sexual assault, mugging or robbery), accidents (such as traffic or workplace accidents), and witnessing something terrible happen. Mass traumatic events include war, torture, terrorist attacks, mass shootings, and severe weather events (flood, earthquake, hurricane, tsunami, forest and bush fire). Sudden memories of previous events can also cause trauma.

Indirect exposure can also cause trauma, for example witnessing others experience a potentially traumatic event, learning that a potentially traumatic event occurred to someone you know, or repeated or extreme exposure to details of a potentially traumatic events, or multiple potentially traumatic events.

Trauma: an emotional response to a potentially traumatic event. Immediate trauma responses can include shock and denial. Longer term reactions include unpredictable emotions, flashbacks, strained relationships and physical symptoms like headaches or nausea.

Abuse: mistreatment that occurs between people (interpersonal trauma), e.g. emotional, physical or sexual abuse including family violence, child abuse, elder abuse, torture and war crimes.

Professional help: help given by a broad range of relevantly trained health professionals. This could include a mental health professional, GP/family doctor, hospital emergency staff, ambulance officer or paramedic. In this survey these professionals will be called professional helpers.

Emergency services: services that respond to and deal with emergencies when they occur, e.g. emergency medical services (ambulance) or law enforcement (the police).

Instructions (continued)

Please complete the questionnaire by rating each statement according to how important you believe it is for inclusion in the guidelines for providing mental health first aid to a person after a potentially traumatic event.

Please keep in mind that the guidelines will be used by the general public. The statements need to be rated according to their importance for someone WITHOUT a counselling or clinical background. **Please note that we do not seek to replicate areas covered by other existing guidelines.** The final guidelines will direct first aiders to the appropriate mental health first aid guidelines, e.g. depression, suicidal thoughts and panic attack if required (mental health first aid guidelines can be found here).

The majority of statements in the questionnaire pertain to both adults and adolescents. There is also a small section with statements that are additional considerations for adolescents and pertain only to assisting an adolescent.

Overview of the questionnaire
Section 1: Background Information
Section 2: Actions to be taken at the site of a potentially traumatic event
Section 3: Talking about the trauma
Section 4: Experiences of Abuse
Section 5: Providing support in the weeks and months following a traumatic experience or a disclosure
Section 6: Adolescents

* 1. What is your name?
(This allows us to determine who has completed the Round 2 survey and is therefore eligible to participate in Round 3- if a third round is necessary. Your name will be deleted from your data when the project is complete).

Background information

This section contains statements about what the first aider needs to know about how someone may react to a potentially traumatic event.

Please rate how important (from 'essential' to 'should not be included') you think it is that each statement be included in the guidelines.

Please keep our definitions in mind when responding to this section. You can access the definitions here.

* 2. The first aider should know the range of symptoms that can occur long-term due to a potentially traumatic event.

- ○ Essential
- ○ Important
- ○ Don't know/depends
- ○ Unimportant
- ○ Should not be included

ROUND 2: Mental Health First Aid Guidelines for helping someone after a potentially traumatic event

Actions to be taken at the site of a potentially traumatic event

This section contains statements about what actions the first aider should take at the site of a potentially traumatic event. Some common examples of situations and events that have the potential to cause trauma include interpersonal violence, accidents, witnessing something terrible happen and mass traumatic events. The items in this section concern how a first aider might provide mental health first aid and do not cover physical first aid.

Please rate how important (from 'essential' to 'should not be included') you think it is that each statement be included in the guidelines.

Please keep our definitions in mind when responding to this section. You can access the definitions here.

* 3. If the first aider does not know the person, they should find out the person's name and use it when talking to them.

- ◯ Essential
- ◯ Important
- ◯ Don't know/depends
- ◯ Unimportant
- ◯ Should not be included

* 4. If the person is already being helped, the first aider should ask the person if they need additional assistance. (New Item)

- ◯ Essential
- ◯ Important
- ◯ Don't know/depends
- ◯ Unimportant
- ◯ Should not be included

* 5. The first aider should find out what the person's immediate needs are (e.g. food, clothing, shelter, medical help or emotional support) and attempt to meet them.

- ◯ Essential
- ◯ Important
- ◯ Don't know/depends
- ◯ Unimportant
- ◯ Should not be included

* 6. The first aider should try to minimise the person's exposure to potentially upsetting sights and sounds, e.g. injured people or flashing lights.

- Essential
- Important
- Don't know/depends
- Unimportant
- Should not be included

* 7. If the person has been a victim of crime, the first aider should consider the possibility that forensic evidence may need to be collected (e.g. evidence on clothing or skin) and should encourage the person to preserve such evidence.

- Essential
- Important
- Don't know/depends
- Unimportant
- Should not be included

* 8. If the person seems confused, the first aider should attempt to orientate the person to the current place or time.

- Essential
- Important
- Don't know/depends
- Unimportant
- Should not be included

* 9. If the person appears overwhelmed or indecisive, the first aider should assist them to make necessary decisions, e.g. about safety.

- Essential
- Important
- Don't know/depends
- Unimportant
- Should not be included

* 10. The first aider should try to keep the person updated about what is happening and what is likely to happen next.

- Essential
- Important
- Don't know/depends
- Unimportant
- Should not be included

* 11. If the first aider thinks that someone is not acting in the person's best interests (e.g. trying to get a media interview or stop them reporting to authorities), the first aider should try to protect the person from this until professionals can step in. (New Item)

- ◯ Essential
- ◯ Important
- ◯ Don't know/depends
- ◯ Unimportant
- ◯ Should not be included

* 12. The first aider should try to stay with the person for as long as the person feels it is needed.

- ◯ Essential
- ◯ Important
- ◯ Don't know/depends
- ◯ Unimportant
- ◯ Should not be included

* 13. If the first aider needs to leave the person, they should make sure someone else is available to stay with the person. (New Item)

- ◯ Essential
- ◯ Important
- ◯ Don't know/depends
- ◯ Unimportant
- ◯ Should not be included

Talking about the trauma

This section contains statements about what the first aider needs to know when talking with the person about their experiences and feelings following a potentially traumatic event, as well as dealing with challenges that may arise in the discussion. These strategies might be used by the first aider when talking with the person at any stage after a potentially traumatic event. They only cover talking about trauma with the person and do not aim to duplicate other mental health first aid guidelines, e.g. depression.

Please rate how important (from 'essential' to 'should not be included') you think it is that each statement be included in the guidelines.

Please keep our definitions in mind when responding to this section. You can access the definitions here.

* 14. The first aider should reassure the person that their reactions are to be **expected** under the circumstances.

- Essential
- Unimportant
- Important
- Should not be included
- Don't know/depends

* 15. The first aider should reassure the person that their reactions are **understandable** under the circumstances. (New Item)

- Essential
- Unimportant
- Important
- Should not be included
- Don't know/depends

* 16. If the person begins a sensitive conversation and the first aider does not think it is the ideal time or place to talk to the person, they should offer to continue the conversation at another time. (New Item)

- Essential
- Unimportant
- Important
- Should not be included
- Don't know/depends

* 17. The first aider should avoid touching the person without their permission, even if touch is usual to their relationship.

- Essential
- Important
- Don't know/depends
- Unimportant
- Should not be included

Talking about the person's feelings

* 18. If the person does not wish to talk to the first aider about how they are feeling, they should encourage the person to seek other supports available to them, e.g. family, friends, support services. (New Item)

- Essential
- Important
- Don't know/depends
- Unimportant
- Should not be included

Talking about the person's experiences

* 19. If the person talks repetitively about the potentially traumatic event, the first aider should listen.

- Essential
- Important
- Don't know/depends
- Unimportant
- Should not be included

Dealing with challenges during the conversation

* 20. If the person seems to be 'spaced out', 'shuts down' or is struggling to communicate, the first aider should talk with the person about other topics. (New Item)

- Essential
- Important
- Don't know/depends
- Unimportant
- Should not be included

* 21. If the person experiences flashbacks, the first aider should ask the person how they wish to be supported when these occur.

- Essential
- Important
- Don't know/depends
- Unimportant
- Should not be included

* 22. If the person experiences a flashback the first aider should tell the person that even though it seems real it is not actually happening.

- Essential
- Important
- Don't know/depends
- Unimportant
- Should not be included

Experiences of abuse

This section contains statements about what the first aider needs to know when talking with the person following a disclosure of abuse. Abuse for the purpose of this survey includes events that occur between people (interpersonal trauma), e.g. emotional, physical or sexual abuse including family violence, child abuse, elder abuse, torture and war crimes.

Please rate how important (from 'essential' to 'should not be included') you think it is that each statement be included in the guidelines.

Please keep our definitions in mind when responding to this section. You can access the definitions here.

* 23. If the person discloses any abuse associated with criminal activity, the first aider should encourage the person to report it to the police, if it is safe to do so.

- ◯ Essential
- ◯ Important
- ◯ Don't know/depends
- ◯ Unimportant
- ◯ Should not be included

* 24. If the first aider reports any criminal activity to the police, they should let the person know beforehand.

- ◯ Essential
- ◯ Important
- ◯ Don't know/depends
- ◯ Unimportant
- ◯ Should not be included

* 25. If the first aider expresses their concerns about signs of physical abuse and the person dismisses them and becomes angry, the first aider should explain they only asked out of concern and will continue to be there if the person needs someone to talk to. (New Item)

- ◯ Essential
- ◯ Important
- ◯ Don't know/depends
- ◯ Unimportant
- ◯ Should not be included

* 26. If the first aider is concerned that the person is at risk of harm from someone else, they should:

	Essential	Important	Don't know/depends	Unimportant	Should not be included
Offer options to keep the person safe, e.g. helping them find alternative accommodation.	◯	◯	◯	◯	◯
Encourage the person to call the police and report the situation.	◯	◯	◯	◯	◯

* 27. If the first aider thinks they need to share any of the information the person has told them in order to keep the person safe, they should tell the person before doing so.

◯ Essential

◯ Important

◯ Don't know/depends

◯ Unimportant

◯ Should not be included

* 28. If the person begins to relate details of the abuse that the first aider finds distressing, the first aider should: (New Item)

	Essential	Important	Don't know/depends	Unimportant	Should not be included
Be honest with the person about this.	◯	◯	◯	◯	◯
Tell the person they do not have the expertise.	◯	◯	◯	◯	◯
Ask the person if they would like assistance finding someone else they can talk to.	◯	◯	◯	◯	◯
Ask the person if they would like to be linked to support services	◯	◯	◯	◯	◯

Providing support in the weeks & months following a potentially traumatic event

This section contains statements about what the first aider needs to know about providing support to the person following a potentially traumatic event. It covers what the first aider can do in the weeks and months following a traumatic event but does not cover ongoing support. These statements are specific to trauma and do not duplicate other mental health first aid guidelines, e.g. depression (mental health first aid guidelines can be found here).

Please rate how important (from 'essential' to 'should not be included') you think it is that each statement be included in the guidelines.

Please keep our definitions in mind when responding to this section. You can access the definitions here.

Being supportive and understanding

* 29. The first aider should tell the person that it is possible to recover from potentially traumatic events.

- ◯ Essential
- ◯ Important
- ◯ Don't know/depends
- ◯ Unimportant
- ◯ Should not be included

Adolescents

This section contains statements about what the first aider needs to know when the person is an adolescent. These are *additional considerations* that only apply to adolescents.

Please rate how important (from 'essential' to 'should not be included') you think it is that each statement be included in the guidelines.

Please keep our definitions in mind when responding to this section. You can access the definitions here.

* 30. If appropriate to the relationship, and with the adolescent's consent, the first aider should contact the adolescent's school about any additional support they may need. (New Item)

- ◯ Essential
- ◯ Important
- ◯ Don't know/depends
- ◯ Unimportant
- ◯ Should not be included

* 31. If the adolescent does not want to talk about what has occurred, the first aider should:

	Essential	Important	Don't know/depends	Unimportant	Should not be included
Encourage the adolescent to talk about their feelings rather than about what has occurred.	◯	◯	◯	◯	◯

Thank you!

Thank you for sharing your expertise and time with us.

If anything in this survey has caused you distress and you would like to talk with someone about it you can contact the appropriate crisis help line for your country. The help line number for your country can be found in the information provided to you by researchers or by visiting this website:
http://www.cocoonais.com/mental-health-hotlines-worldwide/
