# Supplementary material for: Offering mental health first aid to a person after a potentially traumatic event: a Delphi study to redevelop the 2008 guidelines
Source: BMC Psychol. 2020 Oct 6;8:105. doi: 10.1186/s40359-020-00473-7 (PMC7542436; doi:10.1186/s40359-020-00473-7)
Supplement: Supplementary file 3 — Additional file 3. Round 3 Survey. Full survey participants completes in round three. Includes introduction given to participants, consent section and all survey items. [file 40359_2020_473_MOESM3_ESM.pdf]

ROUND 3: Mental Health First Aid Guidelines for helping someone after a potentially traumatic event

Aim of this research

The aim of this current research project is to update the mental health first aid guidelines for how a member of the public should give assistance to a person after a potentially traumatic event. The previously developed mental health first aid guidelines for trauma were created in 2008.

Your role in Round 3

Your task is to rate the statements presented in this questionnaire according to how important you believe they are for providing guidance to adults assisting a person after a potentially traumatic event.

How this questionnaire was developed

Statements in this questionnaire are all items for re-rating. These are items that were new in Round 2 and did not receive a level of consensus that was either high enough to be clearly endorsed or low enough to be clearly rejected.

It is important to note that we do not necessarily agree with these statements; we have included them because we do not believe that we should decide what the best practice is in this area. Rather, we have invited you to be a member of the expert panel to help develop a set of guidelines that reflect current expert opinion.

Instructions

Please complete the questionnaire by rating each statement according to how important you believe it is for inclusion in guidelines for how a member of the public should give assistance to a person after a potentially traumatic event. This questionnaire should take approximately 3-5 minutes to complete. If you are interrupted, you can complete the survey at a later time. Your answers are saved when you click ‘Next’ at the bottom of a page. This marks your page and you can begin again on the next page. **Please be aware that once you have logged on and started responding you must complete the questionnaire on this same computer.**

Your privacy
We intend to protect your anonymity and the confidentiality of your responses to the fullest possible extent, within the limits of the law. Please note that due to the small number of participants anonymity cannot be guaranteed. Any data we collect from you will be held under password protection and not given to others. We are interested in the consensus views of the panel, rather than the views of individual members. We will only present the results in statistical summary form. We occasionally use participant quotes in published journal articles. When this occurs we do not publish any identifying information with the quote.

Data will be kept securely for a period of 5 years after the last publication based on this data. It will not be deleted until all continued interest in the information ceases.

If you have concerns about the project
This research project has been approved by the Human Research Ethics Committee of The University of Melbourne. If you have any concerns or complaints about the conduct of this research project, which you do not wish to discuss with the research team, you should contact the Manager, Human Research Ethics, Research Ethics and Integrity, University of Melbourne, VIC 3010. Tel: +61 3 8344 2073 or Email: HumanEthics-complaints@unimelb.edu.au. All complaints will be treated confidentially. In any correspondence please provide the name of the research team or the name or ethics ID number of the research project.

The ethics number for this research project is 1851764.1

For more information
You received a Plain Language Statement when you expressed interest in this project. Please refer to this for more details about this study. You may also contact Research Officer, Kathryn Chalmers if you require further information: kchalmers1@unimelb.edu.au.

It is important for you to know that participation in this study is completely voluntary. You are not under any obligation to participate and you can withdraw at any time. Submitting your questionnaire is an indication of your understanding of this and your consent to participate in the study.

We would like to thank you for your time and effort and encourage you to provide us with feedback on this process.

Best Wishes,

The University of Melbourne and Mental Health First Aid Australia Research Team

ROUND 3: Mental Health First Aid Guidelines for helping someone after a potentially traumatic event

Instructions

Definitions used in this survey:

Mental health first aid is the help provided to a person who is developing a mental health problem, experiencing a worsening of an existing mental health problem or in a mental health crisis. The first aid is given until appropriate professional help is received or the crisis resolves.

The person: someone who is experiencing extreme distress due to a potentially traumatic event.

The first aider: a concerned family member, friend, work colleague or community member, who provides help to a person experiencing extreme distress due to a potentially traumatic event.

Potentially traumatic event: powerful and distressing experiences that are usually life threatening or pose a significant threat to a person’s physical or psychological wellbeing.

Many events, past and present, have the potential to be traumatic and not all events commonly perceived as traumatic will cause extreme distress for an individual. Some common examples of events that have the potential to cause trauma include interpersonal violence (including family violence, child abuse, elder abuse, physical or sexual assault, mugging or robbery), accidents (such as traffic or workplace accidents), and witnessing something terrible happen. Mass traumatic events include war, torture, terrorist attacks, mass shootings, and severe weather events (flood, earthquake, hurricane, tsunami, forest and bush fire). Sudden memories of previous events can also cause trauma.

Indirect exposure can also cause trauma, for example witnessing others experience a potentially traumatic event, learning that a potentially traumatic event occurred to someone you know, or repeated or extreme exposure to details of a potentially traumatic events, or multiple potentially traumatic events.

Trauma: an emotional response to a potentially traumatic event. Immediate trauma responses can include shock and denial. Longer term reactions include unpredictable emotions, flashbacks, strained relationships and physical symptoms like headaches or nausea.

Abuse: mistreatment that occurs between people (interpersonal trauma), e.g. emotional, physical or sexual abuse including family violence, child abuse, elder abuse, torture and war crimes.

Professional help: help given by a broad range of relevantly trained health professionals. This could include a mental health professional, GP/family doctor, hospital emergency staff, ambulance officer or paramedic. In this survey these professionals will be called professional helpers.

Emergency services: services that respond to and deal with emergencies when they occur, e.g. emergency medical services (ambulance) or law enforcement (the police).

Instructions (continued)

Please complete the questionnaire by rating each statement according to how important you believe it is for inclusion in the guidelines for providing mental health first aid to a person after a potentially traumatic event.

Please keep in mind that the guidelines will be used by the general public. The statements need to be rated according to their importance for someone WITHOUT a counselling or clinical background. **Please note that we do not seek to replicate areas covered by other existing guidelines.** The final guidelines will direct first aiders to the appropriate mental health first aid guidelines, e.g. depression, suicidal thoughts and panic attack if required (mental health first aid guidelines can be found here).

The majority of statements in the questionnaire pertain to both adults and adolescents. There is also a small section with statements that are additional considerations for adolescents and pertain only to assisting an adolescent.

Overview of the questionnaire
Section 1: Actions to be taken at the site of a potentially traumatic event
Section 2: Talking about the trauma
Section 3: Experiences of Abuse

* 1. What is your name?
(This allows us to provide you with the final report. Your name will be deleted from your data when the project is complete).

Actions to be taken at the site of a potentially traumatic event

This section contains statements about what actions the first aider should take at the site of a potentially traumatic event. Some common examples of situations and events that have the potential to cause trauma include interpersonal violence, accidents, witnessing something terrible happen and mass traumatic events. The items in this section concern how a first aider might provide mental health first aid and do not cover physical first aid.

Please rate how important (from 'essential' to 'should not be included') you think it is that each statement be included in the guidelines.

Please keep our definitions in mind when responding to this section. You can access the definitions here.

* 2. If the person is already being helped, the first aider should ask the person if they need additional assistance.

- ◯ Essential
- ◯ Important
- ◯ Don't know/depends
- ◯ Unimportant
- ◯ Should not be included

* 3. If the first aider needs to leave the person, they should make sure someone else is available to stay with the person.

- ◯ Essential
- ◯ Important
- ◯ Don't know/depends
- ◯ Unimportant
- ◯ Should not be included

Talking about the trauma

This section contains statements about what the first aider needs to know when talking with the person about their experiences and feelings following a potentially traumatic event, as well as dealing with challenges that may arise in the discussion. These strategies might be used by the first aider when talking with the person at any stage after a potentially traumatic event. They only cover talking about trauma with the person and do not aim to duplicate other mental health first aid guidelines, e.g. depression.

Please rate how important (from 'essential' to 'should not be included') you think it is that each statement be included in the guidelines.

Please keep our definitions in mind when responding to this section. You can access the definitions here.

* 4. The first aider should reassure the person that their reactions are understandable under the circumstances.

- ◯ Essential
- ◯ Unimportant
- ◯ Important
- ◯ Should not be included
- ◯ Don't know/depends

Talking about the person's feelings

* 5. If the person does not wish to talk to the first aider about how they are feeling, they should encourage the person to seek other supports available to them, e.g. family, friends, support services.

- ◯ Essential
- ◯ Unimportant
- ◯ Important
- ◯ Should not be included
- ◯ Don't know/depends

Experiences of abuse

This section contains statements about what the first aider needs to know when talking with the person following a disclosure of abuse. Abuse for the purpose of this survey includes events that occur between people (interpersonal trauma), e.g. emotional, physical or sexual abuse including family violence, child abuse, elder abuse, torture and war crimes.

Please rate how important (from ‘essential’ to ‘should not be included’) you think it is that each statement be included in the guidelines.

Please keep our definitions in mind when responding to this section. You can access the definitions here.

* 6. If the person begins to relate details of the abuse that the first aider finds distressing, the first aider should be honest with the person about this.

○ Essential
○ Important
○ Don't know/depends
○ Unimportant
○ Should not be included

ROUND 3: Mental Health First Aid Guidelines for helping someone after a potentially traumatic event

Thank you!

That is the end of the third and final survey! Thank you very much for your contribution across all three rounds. We are sincerely grateful to have your input into the development of such an important resource.

If anything in this survey has caused you distress and you would like to talk with someone about it you can contact the appropriate crisis help line for your country. The help line number for your country can be found in the information provided to you by researchers or by visiting this website:
http://www.cocoonais.com/mental-health-hotlines-worldwide/
