# Supplementary material for: Offering mental health first aid to a person after a potentially traumatic event: a Delphi study to redevelop the 2008 guidelines
Source: BMC Psychol. 2020 Oct 6;8:105. doi: 10.1186/s40359-020-00473-7 (PMC7542436; doi:10.1186/s40359-020-00473-7)
Supplement: Supplementary file 5 — Additional file 5. Table 1 Participant characteristics. Tables presenting participant demographics. [file 40359_2020_473_MOESM5_ESM.docx]

**Table 1: Participant characteristics**

|  | **Age range (years)** | **Median age (years)** | **# Female** | **# Male** | **# Identifies with another term** | **# Australians** | **# Europeans** | **# North Americans** |
| --- | --- | --- | --- | --- | --- | --- | --- | --- |
| Professionals (n=28) | 33-71 | 52.0 | 24 | 4 | 0 | 10 | 11 | 7 |
| Consumer Advocates (n=26) | 24-81 | 52.5 | 15 | 8 | 3 | 12 | 11 | 3 |
